# Fluorinated cyclohexanes: Synthesis of amine building blocks of the all-*cis* 2,3,5,6-tetrafluorocyclohexylamine motif

**DOI:** 10.3762/bjoc.13.72

**Published:** 2017-04-19

**Authors:** Tetiana Bykova, Nawaf Al-Maharik, Alexandra M Z Slawin, David O'Hagan

**Affiliations:** 1 School of Chemistry, University of St Andrews, North Haugh, St Andrews, KY16 9ST, UK

**Keywords:** all-*cis* tetrafluorocyclohexane motif, deoxofluorination reactions, fluorinated amines, fluorinated cyclohexanes

## Abstract

This paper reports the synthesis of three amine stereoisomers **5a–c** of the tetrafluorocyclohexyl ring system, as building blocks for discovery chemistry programmes. The synthesis starts from a Birch reduction of benzonitrile, followed by an in situ methyl iodide quench. The resultant 2,5-cyclohexadiene was progressed via double epoxidations and then hydrofluorination ring opening reactions. The resultant fluorohydrin moieties were then converted to different stereoisomers of the tetrafluorocyclohexyl ring system, and then reductive hydrogenation of the nitrile delivered three amine stereoisomers. It proved necessary to place a methyl group on the cyclohexane ring in order to stabilise the compound against subsequent HF elimination. The two all-*cis* tetrafluorocyclohexyl isomers **5a** and **5b** constitute facially polarized cyclohexane rings, with fluorines on the electronegative face and hydrogens on the electropositive face.

## Introduction

**Figure 1:**
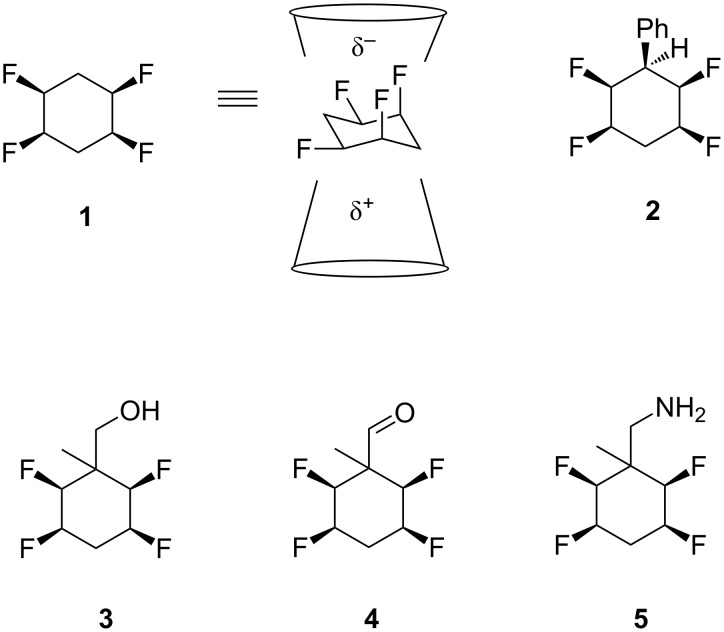
The derivatives of all-*cis*-2,3,5,6-tetrafluorocyclohexane.

The all-*cis*-2,3,5,6-tetrafluorocyclohexane **1** ring has been introduced recently as a polarized cyclohexane ring and it has been a focus of our research group to elaborate new building blocks that enable the introduction of this motif into organic discovery programmes (Figure 1) [1–3]. The cyclohexane ring has four C–F bonds on one face, two of which are aligned 1,3-diaxial. Due to the particularly polar nature of the C–F bond, the alignment of those two bonds results in a large molecular dipole moment [4–6]. For the parent cyclohexane ring **1** the magnitude of the molecular dipole is 5.2 D [2]. The motif has the unique property of inducing facial polarity to the ring system [2,7]. The nature of the interaction of this ring system with protein targets remains to be examined, and its incorporation into organic materials is in its infancy. Access to this motif by the wider research community requires that a range of building blocks be prepared. It has proven relatively straightforward to prepare the phenyl derivative **2** and then subsequent elaboration to a range of functionalized analogues by standard electrophilic aromatic substitution reactions (Figure 1) [1,8–10]. A greater challenge involved the preparation of the tetrafluorocyclohexane ring derivatives without the attached aromatic ring. We have recently demonstrated that both diastereoisomers of alcohols **3** and their corresponding aldehydes **4** (Figure 1), could be prepared after a Birch reduction on benzoic acid, quenching with methyl iodide, and then subsequent conversion of the cyclohexadiene product to a tetrafluorocycloheane motif [11]. The methyl group was a design feature to block hydrogen fluoride elimination from the position alpha to the aldehyde. The diastereoisomers of all *cis-*tetrafluorocyclohexane aldehydes **4** were used successfully in Ugi multicomponent reactions [11–12]. In this paper we report the preparation of amines of this series starting from a Birch reduction on benzonitrile, and with a similar methyl iodide quench, to generate amines **5**, which are stable to hydrogen fluoride elimination.

## Results and Discussion

**Scheme 1:**
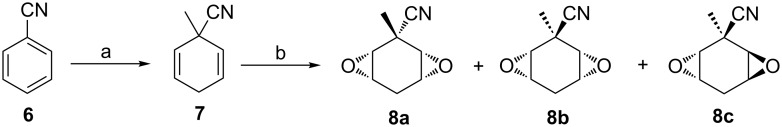
Reagents and conditions: a) Li (2.5 equiv), NH_3_, *t-*BuOH (1 equiv), MeI (2 equiv), 3 h, −78 °C, 18 h, rt, 31%; b) *m*CPBA (4.9 equiv), DCM, 48 h, 35 °C, 18% (**8a**), 35% (**8b** and **8c**), ratio **8a**:**8b**:**8c**: 10:15:13.

The Birch reduction of benzonitrile **6** followed by in situ methylation with iodomethane generated cyclohexadiene **7** as previously described [13–14]. Cyclohexadiene **7** was then subjected to a double epoxidation protocol using *m*CPBA [1,11,15–16]. This generated three diastereoisomers of **8**, two of which have the *meso* diepoxides *syn* and *anti* in relation to the nitrile functional group (**8a** and **8b**) and a racemic **8c** diepoxide with *trans* configuration. These isomers were generated in a ratio of 10:15:13 (**8a**:**8b**:**8c**) (Scheme 1) [15]. Diepoxide **8a** was isolated by chromatography (18% yield), however, diepoxides **8b** and **8c** (35%) could not be separated, and therefore were taken as a mixture of isomers to the next step in the reaction sequence.

**Scheme 2:**
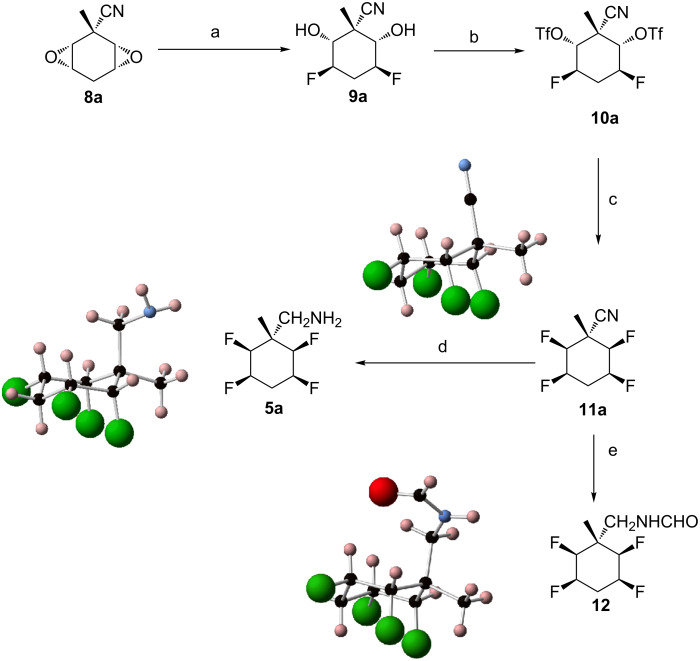
Reagents and conditions: a) Et_3_N·3HF (8 equiv), 18 h, 140 °C; b) Tf_2_O (4 equiv), pyridine, 1 h, 0 °C, 3 h, rt, 30%; c) Et_3_N·3HF (10 equiv), 4 days, 120 °C, 30%; d) NaBH_4_ (10 equiv)/NiCl_2_·6H_2_O (5 equiv), MeOH, 1 h, 0 °C, 18 h, rt, 50%; e) 10% Pd/C (10 mol %), H_2_, Et_3_N/CHOOH: molar ratio 1:37, THF, 18 h, rt, 78%.

Treatment of **8a** with Et_3_N·3HF at 140 °C resulted in its full conversion to the hydrofluorinated ring-opened diol **9a** as a single regioisomer, a product which was treated directly with triflic anhydride in pyridine, to generate ditriflate **10a** in a 30% yield over two steps (Scheme 2). We have found that related treatments with the more acidic Olah’s reagent (HF·pyridine) were less satisfactory generating product mixtures [2,17].

**Scheme 3:**
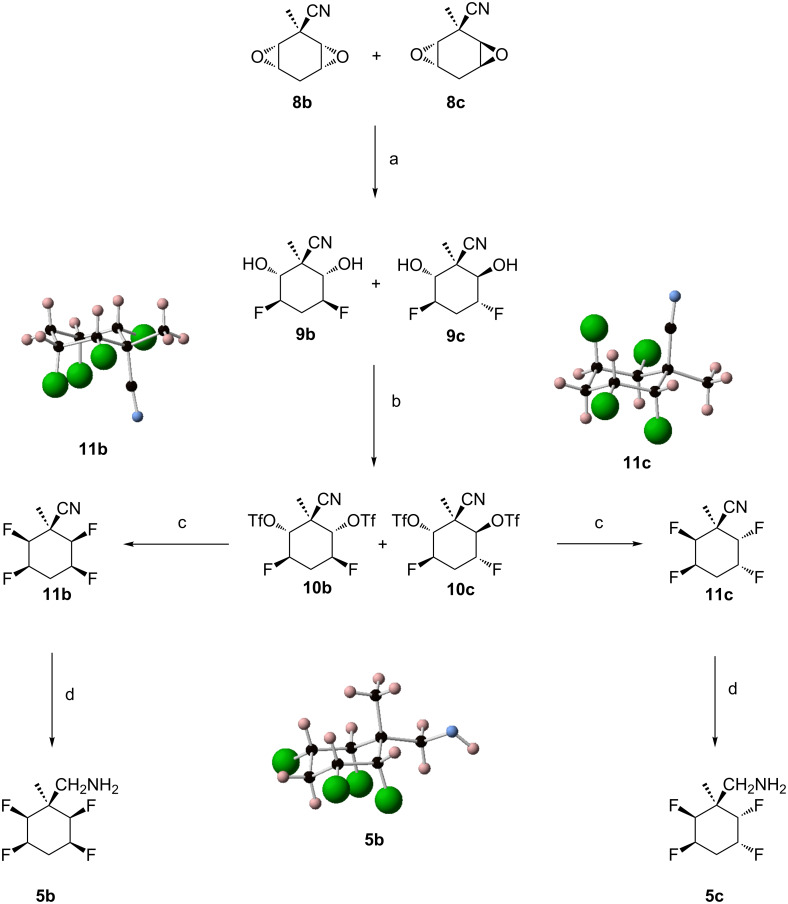
Reagents and conditions: a) Et_3_N·3HF (8 equiv), 18 h, 140 °C; b) Tf_2_O (4 equiv), pyridine, 1 h, 0 °C, 3 h, rt, 40% (**10b**), 15% (**10c**); c) Et_3_N·3HF (10 equiv), 4 days, 120 °C, 51% (**11b**), 31% (**11c**); d) NaBH_4_ (10 equiv)/NiCl_2_·6H_2_O (5 equiv), MeOH, 1 h, 0 °C, 18 h, rt, 65% (**5b**), 23% (**5c**).

Treatment of **10a** with Et_3_N·3HF at 120 °C generated the tetrafluorocyclohexane **11a,** the structure of which was confirmed by X-ray crystallography (Scheme 2). Although the conversion of **10a** to **11a** was high as judged by ^19^F NMR, the isolated yield was modest as the compound was volatile and sublimed easily under reduced pressure. Hydrogenation of **11a** over 10% Pd/C in ethyl acetate gave the desired amine **5a** but in very low yield (0–15%) [18–20]. The structure of **5a** was confirmed by X-ray crystallography (Scheme 2). Adding a few drops of formic acid and triethylamine (molar ratio 37:1) to the hydrogenation, furnished formamide **12** in 78% yield, a compound also confirmed by X-ray crystallography [20]. Ultimately treatment of nitrile **11a** with nickel boride generated in situ from nickel chloride and sodium borohydride, resulted in its full reduction to amine **5a** in 50% yield (Scheme 2) [21–22]. The analogous protocol was then applied to diastereoisomers **8b** and **8c** as illustrated in Scheme 3. Fluorination of the isomer mixture **8b/8c** with Et_3_N·3HF at 140 °C gave **9b** and the racemic **9c** as an inseparable mixture. Triflation of this product mixture generated **10b** and **10c**, isomeric products which could now be separated by chromatography. Finally fluorination of **10b** and **10c** in separate reactions with Et_3_N·3HF at 120 °C furnished nitrile **11b** (51% yield) and **11c** (31% yield) respectively.

The structure and stereochemistry of both products were unambiguously confirmed by X-ray structure analysis (Scheme 3). Reduction of **11b** with nickel boride delivered amine **5b** in 65% yield as a crystalline solid and an analogous reduction of **11c** generated the racemic amine **5c** as a colourless liquid. The structure of **11b** was confirmed by X-ray structure analysis (Scheme 3).

Amines **5a** and **5b** were reacted with terephthaloyl chloride as a means of preparing higher order, *bis*-amide systems to explore intermolecular packing of the cyclohexane rings in the solid state. To extend the study a comparison was also made with the ester derivative from the previously prepared alcohol **3a** [11].

**Scheme 4:**
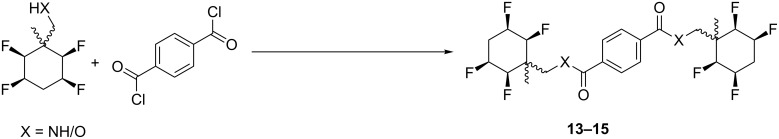
Reagents and conditions: a) terephthaloyl chloride (1 equiv), Et_3_N (4 equiv), DMAP (20 mol %), DCM, 18 h, rt, 79% (**13**), 48% (**14**), 88% (**15**).

Treatment of 2.1 equivalents of 2,3,5,6-tetrafluorocyclohexane derivatives **5a**, **5b** and **3a** with 1 equivalent of terephthaloyl chloride, in the presence of triethylamine (4 equiv) and DMAP resulted in *bis*-systems **13**, **14** and **15** in 79%, 48%, and 88% yields respectively (Scheme 4) [23–24]. All of the above compounds were found to be solid materials with low solubility. However suitable crystals were obtained from acetone/acetonitrile for X-ray structure analysis.

**Figure 2:**
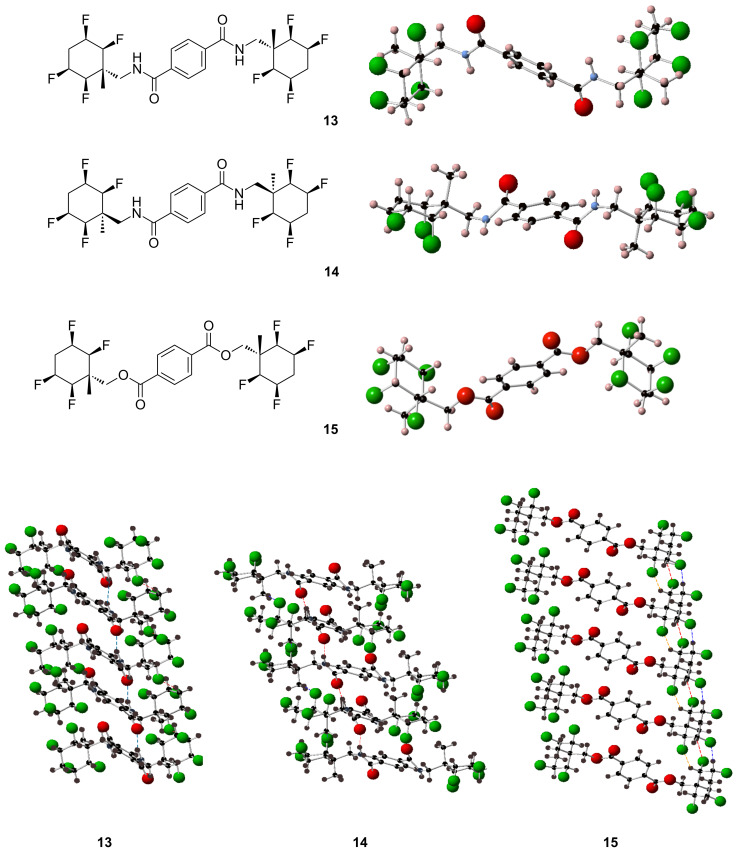
X-ray structures and crystal packing of compounds **13**, **14** and **15**.

The X-ray structures of **13**, **14** and **15** are illustrated in Figure 2. It was assumed that the facial polarity of the tetrafluorocyclohexane rings would be apparent in the molecular ordering in the solid state, with intercalations between negative fluorous faces and positive protic faces of the rings. This was the case as can be seen from the packing structures in Figure 2. For compounds **13** and **14** intermolecular hydrogen bonding between the amide groups also contributes to this stacking structure but for compound **15**, there are no amides to stabilize such a structure, and the facially polarized cyclohexane rings presumably form the strongest intermolecular interactions in this case (Figure 2).

## Conclusion

In summary we report a method for the preparation of 2,3,5,6-tetrafluorocyclohexane amines **5a**, **5b** and **5c**, through a synthesis sequence starting from the Birch reduction of benzonitrile. *Bis*-compounds **13**–**15** were readily prepared from amines **5a**, **5b** and an alcohol **3a** in reactions with terephthaloyl chloride. The structures of which indicate arrangements in the solid state consistent with electrostatic ordering of the cyclohexane rings. These polarized cyclohexyl derivatives should prove valuable as potential building blocks in drug discovery and agrochemistry research programs [3,25].

## Supporting Information

File 1 Experimental part.
