# Supplementary material for: Fluorinated cyclohexanes: Synthesis of amine building blocks of the all-cis 2,3,5,6-tetrafluorocyclohexylamine motif
Source: Beilstein J Org Chem. 2017 Apr 19;13:728–33. doi: 10.3762/bjoc.13.72 (PMC5405691; doi:10.3762/bjoc.13.72)

Supporting Information

for

Fluorinated cyclohexanes: Synthesis of amine building blocks of the all-*cis* 2,3,5,6-tetrafluorocyclohexylamine motif

Tetiana Bykova, Nawaf Al-Maharik, Alexandra M. Z. Slawin and David O'Hagan*

Address: School of Chemistry, University of St Andrews, North Haugh, St Andrews, KY16 9ST, UK.

Email: David O'Hagan - do1@st-andrews.ac.uk

*Corresponding author

Experimental part

Table of Contents

1. GENERAL INFORMATION S2

2. PROCEDURE AND ANALYTICAL DATA S3

3. NMR SPECTRA S9

1. General information

^{1}H, ^{13}C and ^{19}F NMR spectra were recorded on Bruker Avance or Avance III spectometers at 500 MHz (^{1}H NMR), 125 MHz (^{13}C NMR) and 470 MHz (^{19}F NMR). Chemical shifts data were reported as δ in units of parts per million relative to residual solvent and were referenced to the internal solvent, where appropriate. Coupling constants were reported in Hertz (Hz). Analytical thin layer chromatography (TLC) was carried out on Merck silica gel 60 F254 glass-supported plates. Visualisation was by absorption of UV light (λ_{max} 253 or 365 nm), or by thermal development after dipping in an aqueous solution of potassium permanganate, potassium carbonate and sodium hydroxide. Flash column chromatography was carried out on Merck Geduran silica gel 40–63 micron, eluting with solvents as supplied, under a positive pressure of compressed air. Melting points were recorded on an Electrothermal 9100 melting point apparatus and are uncorrected. Electrospray mass spectra were recorded at the University of St Andrews Mass Spectrometry facility on either a Micromass LC TOF spectrometer or a ThermoFisher Orbitrap Excalibur spectrometer from solutions of the analyate in methanol. Additional spectra were obtained at the EPSRC National Mass Spectrometry Service Centre at Swansea using a ThermoFisher Orbitrap LQT XL spectrometer fitted with an ASAP solids probe operating in atmospheric pressure chemical ionisation (APCI) mode.

2. Procedure and analytical data

Following a previously reported method, liquid ammonia was added to a three necked flask cooled to −78 °C, followed by an addition of lithium (2.40 g, 294 mmol) in small portions. A solution of benzonitrile **6** (12 mL, 117.6 mmol) in THF (120 mL) and *tert*-butyl alcohol (11.2 mL, 117.6 mmol) was added dropwise to the reaction mixture. After 3 h of stirring at the following temperature, MeI (14.8 mL, 235 mmol) was added dropwise to the reaction. Ammonia was evaporated overnight followed by an addition of water (400 mL) and NH_4Cl (30 mg), the mixture was extracted three times with diethyl ether. The combined organic phases were dried over $MgSO_4$, filtered, concentrated under reduced pressure, resulting in **7**, 4.38 g, 37 mmol, 31% yield as colourless oil.

Methyl-dioxatricyclooctane-carbonitrile 8a, 8b and 8c:

*m*CPBA (70%) (36 g, 148 mmol) was added to a solution compound **7** (3.6 g, 30 mmol) in DCM (400 mL) at rt. The reaction was allowed to warm to 35 °C and stirred for 24 h. The reaction mixture was then washed with aq. K_2CO_3 (10%) and the aqueous layer was then extracted with DCM (200 mL × 3). The combined organic layers were dried over Na_2SO_4, filtered and concentrated to give a colourless crystalline solid, with three diastereoisomers of diepoxide (**8a**, **8b** and **8c**) observed on ^{1}H NMR spectrum in a ratio of 10: 15: 13.

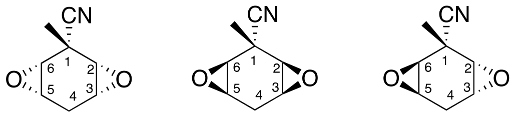

8a 8b 8c

Crude product was then purified by silica gel column chromatography (ethyl acetate/ petroleum ether = 9/ 1, 7.5/ 2.5) giving the desired compounds in two sets of fractions, compounds **8b** and **8c as a mixture** (Rf = 0.74 at ethyl acetate/ petroleum ether = 1/ 1) (1.59 g, 10.5 mmol, 35%); **8b** (major): ^{1}H NMR (400 MHz, CDCl3): δ 3.34-3.17 (4H, m, H-2, 3, 5, 6), 2.83 (1H, d, *J* = 17.5, H-4a), 2.51-2.31 (1H, m, H-4b), 1.67 (3H, s, CH_3); **8c** (minor): ^{1}H NMR (400 MHz, CDCl3): δ 3.34-3.17 (2H, m, H-3, 5), 3.03-2.97 (2H, m, H-2, 6), 2.51-2.31 (1H, m, H-4a,b) 1.80 (3H, s, CH_3), followed by epoxide **8a** (Rf = 0.15 at ethyl acetate/ petroleum ether = 1/ 1) (0.82 g, 5.4 mmol, 18%); ^{1}H NMR (500 MHz, $CDCl_3$) δ 3.31- 3.20 (2H, m, H-3, H-5), 3.16 (1 H, d, *J* = 3.7, H-1, 6), 2.87 (1H, d, *J* = 17.4, H-4a), 2.27 (1H, dt, *J* = 17.4, 2.9, H-4b), 1.65 (3H, s, CH_3); ^{13}C NMR (101 MHz, $CDCl_3$) 119.8 (CN), 54.1 (C-2, 6), 50.1 (C-3, 5), 33.4(C-1), 22.2 (C-4), 20.7 (CH_3).

2-Cyano-4,6-difluoro-2-methylcyclohexane-1,3-diyl bis(trifluoromethanesulfonate 10a

In a dry Teflon flask, $Et_3N.3HF$ (6.8 mL, 42.4 mmol) was added to diepoxide **8a** (0.8 g, 5.3 mmol) at rt. After 12 h of stirring at 140 °C, the mixture was cooled to rt, poured into $NaHCO_3$ (100 mL) and extracted with dichloromethane (100 mL × 3). The combined organic layers were dried over $MgSO_4$, filtered, concentrated under reduced pressure resulting in 0.71g of yellow solid which was taken as a crude to the next stage, **9a**: ^{1}H NMR (400 MHz, $CDCl_3$) δ 4.93-4.49 (2H, m, H-3, 5), 3.53 (2H, dd, *J*-10.7, 9.1, H- 2, 6), 2.79-2.65 (1H, m, H-4a), 1.95-1.78 (1H, m, H-4b), 1.70 (3H, s, CH_3); ^{19}F NMR (376 MHz, $CDCl_3$) δ -191.4.

Trifluoromethanesulfonic anhydride (3.7 mL, 21 mmol) was slowly added to crude product in pyridine (20 mL) at 0°C and allowed gradually to warm up to rt. After 18 h stirring at rt, the reaction mixture was quenched with a mixture of water (100 mL) and copper sulphate (2 mL) followed by extraction with DCM (100 mL x 3). The combined organic phases were dried over Na_2SO_4, filtered and concentrated under reduced pressure. The product was purified by means of column chromatography (petroleum ether/ethyl acetate 7:3) to offer **10a** as a white crystalline solid (722 mg, 1.59 mmol, 30%); M.p. = 168-169 °C; ^{1}H NMR (400 MHz, $CDCl_3$) δ 5.14-4.91 (2H, m, H-3, 5), 4.74 (2H, t, *J* = 9.1 Hz, H-2, 6), 3.20-2.88 (1H, m, H-4a), 2.17-2.00 (1H, m, H-4b), 1.79 (3H, s, CH_3); ^{19}F NMR (376 MHz, $CDCl_3$) δ -72.7 (d, *J* = 14.2, Hz, CF_3), -189.7 (q, *J* = 12.7 Hz, CHF); ^{13}C NMR (126 MHz, $CDCl_3$) δ 118.3 (q, *J* = 320.0 Hz, CF_3), 114.2 (CN), 84.9 (dd, J = 189.2, 14.7 Hz, CHF), 84.3 (d, J = 20.8 Hz, CHOTf), 41.7 (t, *J* = 6.5 Hz, C-1), 31.5 (t, *J* = 21.9 Hz, C-4), 19.9 (CH_3) ; FTMS (ESI^+) *m/z* calcd for ([M]+ Na^+) 477.9641; found 477.9638; IR: v_{max}/cm^{-1} 2960 (C-H), 2873 (C-H), 2360 (C≡N), 1724, 1417 (C-C).

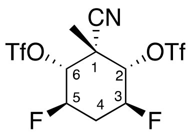

2-Cyano-4,6-difluoro-2-methylcyclohexane-1,3-diyl bis(trifluoromethanesulfonate 10b and 10c:

Following the analogous method as described in synthesis of **10a**, the mixture of the diepoxides **8b** and **8c** (1.5 g, 9.9 mmol) been fluorinated resulting in 1.35 g of yellow solid resulting in a mixture of **9b** and **9c,** which been taken for the next stage without further purification, **9b** (major): ^{1}H NMR (400 MHz, $CDCl_3$) δ 5.13-4.70 (2H, m, H-2, 6), 4.69-4.31 (2H, m, H-3, 5), 2.77 (1H, dd, *J*= 75.6, 4.0, H-4a), 2.54-2.17 (1H, m, H-4b); ^{19}F NMR (376 MHz, $CDCl_3$) -192.15 (s); **9b** (minor): ^{1}H NMR (400 MHz, $CDCl_3$) δ 5.13-4.70 (2H, m, H-2, 6), 4.69-4.31 4.34-3.90 (2H, m, H-3, 5), 2.54-2.17 (2H, m, H-4a, b); ^{19}F NMR (376 MHz, $CDCl_3$) δ -187.6 (s, br), -190.5 (s, br). Following triflation reaction, on purification using column chromatography (petrol ether/ ethyl acetate 95/5) furnishing **10b** (1.82 g, 4.01 mmol, 40%)**;** M.p. = 144-145 °C; ^{1}H NMR (400 MHz, $CDCl_3$) δ 5.16 (2H, dd, *J* = 9.6, 8.6 Hz, H-2, 6), 4.84 (2H, ddd, *J* = 46.6, 11.0, 8.7, Hz, H-3, 5), 2.98-2.83 (1H, m, H-4a), 2.31-2.07 (1H, m, H-4b), 1.63 (3H, s, CH_3); ^{19}F NMR (376 MHz, $CDCl_3$) δ -73.0 (d, *J* = 9.1 Hz, CF_3), -189.6 (s br, CHF); ^{13}C NMR (101 MHz, $CDCl_3$) δ 118.9 (CN), 117.5 (q, *J* = 160.3 Hz), 83.8 (dd, *J* = 188.0, 13.7 Hz, C-3, 5), 82.4 (d, *J* = 21.3 Hz, C-2, 6), 30.7 (t, *J* = 21.8 Hz, C-4), 29.7 (C-1), 14.7 (CH_3); FTMS (ESI^-) *m/z* calcd for ([M]+ Cl^-) 489.9432; found 489.9429; IR: v_{max}/cm^{-1} 2978 (C-H), 2922 (C-H), 2360 (C≡N), 1433 (C-C), 1404 (C-C); followed by **2-cyano-4,6-difluoro-2-methylcyclohexane-1,3-diyl bis(trifluoromethanesulfonate (10c)** as an oily liquid (676 mg, 1.49 mmol, 15%); ^{1}H NMR (400 MHz, $CDCl_3$) δ 5.37-5.02 (3H, m, H-2, 3, 5), 4.87 (1H, t, *J* = 8.3 Hz, H-6), 2.93-2.76 (1H, m, H-4a), 2.30 (1H, ddtd, *J* = 38.2, 15.1, 10.6, 3.0 Hz, H-4b), 1.80 (3H, s, CH_3); ^{19}F NMR (376 MHz, $CDCl_3$) δ -72.9 (d, *J* = 11.4 Hz, CF_3), -73.3 (d, *J* = 1.4 Hz, CF_3), -182.9 (s, CHF), -191.5 (s, CHF); ^{13}C NMR (101

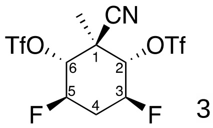

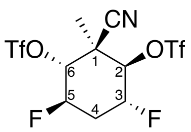

MHz, $CDCl_3$) δ 118.3 (q, *J* = 319.9 Hz, CF_3), 118.2 (q, *J* = 319.8 Hz, CF_3), 114.5 (CN), 85.7 (dd, *J* = 181.8, 11.6 Hz CHF), 85.1 (dd, *J* = 185.3, 2.5 Hz, CHF), 83.7 (d, *J* = 21.1 Hz, CHOTf), 81.7 (d, *J* = 28.0 Hz, CHOTf), 40.7 (d, *J* = 4.6 Hz, C-1), 31.1 (t, *J* = 21.0 Hz, C-4), 20.2 (CH_3); FTMS (ESI^+) *m/z* calcd for ([M]+ Na^+) 477.9641; found 477.9636; IR: v_{max}/cm^{-1} 2908 (C-H), 2341 (C≡N), 1417 (C-C).

2,3,5,6-Tetrafluoro-1-methylcyclohexane-1-carbonitrile (11a)

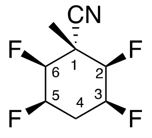

Triflate **10a** (0.7 g, 1.5 mmol) and $Et_3N.3HF$ (2.68 g, 16 mmol) were placed in Teflon round bottom flask equipped with condenser and heated to 120 °C. After the full conversion of the starting material (4 days) the mixture was cooled to rt, poured into $NaHCO_3$ (150 mL) and extracted with DCM (100 mL x 3). Combined organic layers were dried over $MgSO_4$, filtered and concentrated under reduced pressure. The product was purified by column chromatography (Petroleum ether/ ethyl acetate 9/1) to give **11a** (0.88 mg, 0.45 mmol, 30%) as a white crystalline solid, which was found to sublime under reduced pressure; M.p. = 137-138 °C; ^{1}H NMR (400 MHz, $CDCl_3$) δ 5.12-4.70 (4H, m, H-2, 3, 5, 6), 2.72-2.55 (1H, m, H-4a), 2.51-2.43 (1H, m, H-4b), 1.78 (3H, s, CH_3); ^{19}F NMR (376 MHz, $CDCl_3$) δ -194.5 (s br, CHF), -209.0 (s br, CHF); ^{13}C NMR (126 MHz, $CDCl_3$) δ 117.0 (CN), 88.1 (d, *J* = 188.2 Hz, C-2, 6), 85.1 (d, *J* = 192.0 Hz, C-3, 5), 29.7 (C-1) , 26.5 (t, *J* = 15.6 Hz, C-4), 17.5 (CH_3); FTMS (ESI^+) *m/z* calcd for ([M]-CH_4) 179.0358, found 179.0170; IR: v_{max}/cm^{-1} 2362 (C≡N), 1244 (C-F), 1050 (C-F)

2,3,5,6-Tetrafluoro-1-methylcyclohexane-1-carbonitrile (11b)

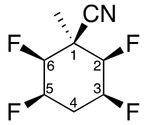

Following the analogous method as described in synthesis of **11a**, triflate **10b** (1.8 g, 3.96 mmol) furnished compound **11b** (394 mg, 2.02 mmol, 51%) white crystalline solid; M.p. = 195 °C; ^{1}H NMR (400 MHz, $CDCl_3$) δ 5.04 (2H, d, *J* = 50.4 Hz, H-2, 6), 4.34 (dd, *J* = 43.2, 21.5 Hz, H-3, 5), 3.01-2.72 (1H, m, H-4a), 2.12-1.85 (1H, m, H-4b), 1.77 (3H, s, CH_3); ^{19}F NMR (376 MHz, $CDCl_3$) δ -198.0 (s br, CHF); ^{13}C NMR (126 MHz, $CDCl_3$) δ 116.5 (CN), 90.2 (d, *J* = 179.6 Hz, C-2, 6), 84.4 (d, *J* = 212.2 Hz, C-3, 5), 31.0 (C-1) , 28.9 (C-4) , 21.3 (CH_3); FTMS (ESI^+) *m/z* calcd for ([M]+ Na^+) 218.0568; found 218.0559; IR: v_{max}/cm^{-1} 2987 (C-H), 2358 (C≡N), 1136 (C-F), 1058 (C-F)

2,3,5,6-Tetrafluoro-1-methylcyclohexane-1-carbonitrile (11c)

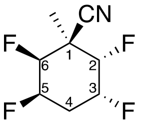

Following the analogous method as described in synthesis of **11a**, triflate **10c** (650 mg, 1.43 mmol) furnished compound **11c** (86 mg, 0.44 mmol, 31%) white crystalline solid; M.p. = 79-80 °C; ^{1}H NMR (400 MHz, $CDCl_3$) δ 5.47-4.87 (3H, m, H-2, 3, 5), 4.68-4.40 (1H, m, H-6), 2.75-2.49 (1H, m, H-4a), 2.43-2.13 (1H, m, H-4b), 1.75 (3H, s, CH_3); ^{19}F NMR (376 MHz, $CDCl_3$) δ -200.2 (s br), -201.0 (s br), -206.5 (s br), -211.6 (s br); ^{13}C NMR (126 MHz, $CDCl_3$) δ 117.3 (CN), 90.5 (d, *J* = 188.9 Hz, C-2), 89.5 (dd, *J* = 180.1, 14.7 Hz, C-3), 86.4 (d, *J* = 190.8 Hz, C-5), 84.7 (d, *J* = 176.6 Hz, C-6), 29.7 (C-1), 28.8 (tt, *J* = 21.1, 5.7 Hz, C-4), 19.5 (CH_3); FTMS (ESI^+) *m/z*

calcd for ([M]+ Na^+) 218.0568; found 218.0562; IR: $ν_{max}/cm^{-1}$ 2991 (C-H), 2330 (C≡N), 1249 (C-F), 1074 (C-F).

2,3,5,6-Tetrafluoro-1-methylcyclohexyl)methanamine (5a)

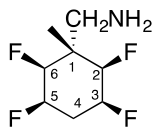

Sodium borohydride (155 mg, 4.1 mmol) was slowly added to a solution of **11a** (80 mg, 0.41 mmol) and nickel(II) chloride hexahydrate (264 mg, 2.05 mmol) in methanol (5 mL) at 0 °C under argon. The reaction mixture was allowed to warm to room temperature and was stirred until disappearance of starting material (TLC). Methanol was evaporated under vacuum, and the resulting crude was diluted with ethyl acetate (100 mL). Then, an aqueous saturated solution of $NaHCO_3$ was added (50 mL). The resulting mixture was filtered through a pad of Celite, and the solution was extracted with ethyl acetate (4 × 50 mL). The organic layer was dried ($MgSO_4$) and concentrated under reduced pressure. The crude mixture was purified using column chromatography to furnish **5a** as a white solid (41 mg, 2.05 mmol, 50%); M.p. = 99-100 °C; ^{1}H NMR (400 MHz, MeOD) δ 5.09-4.98 (2H, m, H-2, 6), 4.58 (2H, dd, *J* = 45.0, 20.5, Hz, H-3, 5), 2.78 (2H, s, CH_2), 2.63-2.43 (1H, m, H-4a), 2.23-2.01 (1H, m, H-4b), 1.31 (3H, t, *J* = 1.6 Hz, CH_3); ^{19}F NMR (376 MHz, MeOD) δ -196.6 (s br) , -209.5 (s br); ^{13}C NMR (101 MHz, MeOD) δ 94.2 (dd, *J* = 185.7, 16.4 Hz), 90.1 (dd, *J* = 183.3, 15.0 Hz). 49.3 (CH_2), 32.1 (C-4), 27.5 (C-1), 16.6 (CH_3); FTMS (ESI^+) *m/z* calcd for ([M]+H) 200.1062; found 200.1051; IR: $ν_{max}/cm^{-1}$ 3520 (N-H), 2972(C-H), 2901 (C-H), 2358, 1734 (N-H), 1417 (C-H); 1215 (C-F)

2,3,5,6-Tetrafluoro-1-methylcyclohexyl)methanamine (5b)

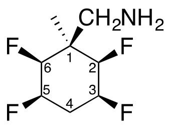

Following the analogous method as described in synthesis of **5a**, tetrafluoro nitrile **11b** (350 mg, 1.79 mmol) provided a tetrafluoro amine **5b** (225 mg, 1.13 mmol, 65%) white crystalline solid; M.p. = 106-107 °C; ^{1}H NMR (500 MHz, MeOD) δ 5.00-4.71 (2H, m, H-2, 6), 4.67 (4H, dm, *J*= 49.8, H-3, 5), 3.09 (2H, s, CH_2-N), 2.61-2.39 (2H, m, H-4), 0.99 (3H, s, CH_3); ^{19}F NMR (377 MHz, MeOD) δ -196.5 (m, AA'XX'), -209.3 (s br); ^{13}C NMR (126 MHz, MeOD) δ 90.6 (dd, *J* = 193.2, 12.9 Hz, C-2, 6), 85.8 (dd, *J* = 181.4, 14.7 Hz, C-3, 5), 43.4 (CH_2NH_2), 27.5 (t, *J* = 21.8 Hz, C-4), 22.7 (C-1) , 14.9 (CH_3); FTMS (ESI^+) *m/z* calcd for ([M]+H) 200.1062; found 200.1053; IR: $ν_{max}/cm^{-1}$ 3510 (N-H), 2972 (C-H), 2900 (C-H), 2357, 1595 (N-H), 1222 (C-F)

(2,3,5,6-Tetrafluoro-1-methylcyclohexyl)methanamine (5c)

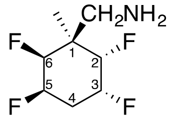

Following the analogous method as described in synthesis of **5a**, tetrafluoro nitrile **11c** (80 mg, 0.41 mmol) provided a tetrafluoro amine **5c** as colourless oil (19 mg, 0.094 mmol, 23%); ^{1}H NMR (400 MHz, MeOD) δ 5.25-4.79 (3H, m, H-2, 3, 5), 4.65 (1H, dd, *J* = 45.1, 26.4, H-6), 2.93 (1H, d, *J* =13.5, CH_{2a}), 2.82 (1H, dt, *J* =13.5, 2.3, CH_{2b}), 2.53-2.11 (2H, m, H-4), 1.17 (3H, s, CH_3); ^{19}F NMR (377 MHz, MeOD) δ -197.9 (s br), -201.8 (s br), -213.4 (s br), -215.4 (s br); ^{13}C NMR (126 MHz, MeOD) δ 93.1 (dd, *J* = 180.5, 5.8 Hz, C-3, 5), 92.4 (dd, *J* = 180.6, 5.8 Hz, C-2, 6), 46.0 (CH_2), 28.7 (t, *J* = 21.2, 6.5 Hz, C-4), 22.8 (C-1), 13.7 (CH_3); FTMS (ESI^+) *m/z* calcd for ([M]+H) 200.1062; found

200.1052; IR: v_{max}/cm^{-1} 3525 (N-H), 2972 (C-H), 2893 (C-H), 2358, 2343, 1558 (N-H), 1069 (C-F).

((2,3,5,6-Tetrafluoro-1-methylcyclohexyl)methyl)formamide (12)

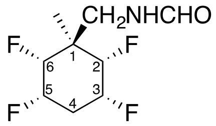

`Following literature described method, to a solution of tetrafluoro nitrile **11a** (110 mg, 0.56 mmol) in THF (5 mL) a mixture of formic acid and triethalamine with molar ratio of 37: 1 (1 mL) was added under argon atmosphere. After the addition of Pd/C (10 mol%), the mixture was degased, flushed three times with H_2, and left stirring under H_2 atmosphere at room temperature until completion (TLC monitoring). The reaction mixture was passed through a pad of celite. The filtrate was concentrated and purified using column chromatography (petrol ether/ diethyl ether 75/ 25) resulting in **12** (99 mg, 0.43 mmol, 78% yield) as a white crystalline solid; M.p.= 146-147 °C; ^{1}H NMR (400 MHz, MeOD) δ 8.16 (1H, s, CHO), 5.05 (d, *J* = 45.3 Hz, H-3, 5), 4.43 (2H, dd, *J* = 43.6, 15.6 Hz, H-2, 6), 3.23 (2H, s br, CH_2-N), 2.55-2.38 (1H, m, H-4a), 2.29-2.08 (1H, m, H-4b), 1.28 (3H, s, CH_3); ^{19}F NMR (376 MHz, MeOD) δ -196.5 (s br), -210.7 (s br); ^{13}C NMR (101 MHz, MeOD) δ 163.1 (CHO), 89.2 (dd, *J* = 188.4, 12.9 Hz, C-3, 5), 85.9 (dd, *J* = 177.1, 13.4 Hz, C-2,6), 43.6 (C-1), 40.6 (CH_2-NH_2), 27.5 (C-4), 13.9 (CH_3); FTMS (ESI^+) *m/z* calcd for ([M]+Na^+) 250.0831; found 250.0825; I IR: v_{max}/cm^{-1} 3246 (N-H), 3064 (OC-H), 1653 (C=O).

Bis-((2,3,5,6-tetrafluoro-1-methylcyclohexyl)methyl)terephthalamide (13)

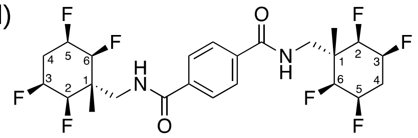

Tetrafluorocyclohexane amine **5a** (30 mg, 0.15 mmol) and DMAP (2 mg, 0.016 mmol) were dissolved in DCM under argon atmosphere followed by addition of Et_3N (30 mg, 0.3 mmol). After cooling to 0 °C, tetephnaloyol chloride (16 mg, 0.075 mmol) was added and the mixture was stirred for 15 min at 0 °C and for further 18 h at room temperature. After the reaction was complete, water was added and the solution was extracted three times with DCM. The combined organic phases were dried over $MgSO_4$ filtered and the solvent was removed under reduced pressure. The product was purified by column chromatography (petroleum ether/ diethyl ether, 9/1) furnishing **13** (31 mg, 0.59 mmol, 79%); M.p. = 292-293°C; ^{1}H NMR (400 MHz, $(CD_3)_2CO$) 8.02 (4H, s, Ar), 5.46-5.12 (4H, m, H-2, 6), 4.67 (4H, dd, *J* = 46.8, 11.7, H-3, 5), 3.46 (4H, s, CH_2), 2.54-2.27 (4H, m, H-4), 1.47 (6H, m, CH_3); ^{19}F NMR (376 MHz, $(CD_3)_2CO$) -195.4 (s br), -210.1 (s br); ^{13}C (126 MHz, $(CD_3)_2CO$) 166.9 (COAr), 136.8 (C(Ar)) , 127.4 (CH(Ar)), 90.6 (dd, *J*=186.9, 12.8, C-2,6), 86.4 (dd, *J* = 194.6, 12.3, C-3, 5), 44.5 (C-1), 42.3 (CH_2) , 27.4 (C-4), 15.5 (CH_3); FTMS (ESI^-) *m/z* calcd for ([M]- H^+) 527.1944; found 527.1946; IR: v_{max}/cm^{-1} 3329 (N-H), 2953 (C-H), 1639 (C=O).

Bis-((2,3,5,6-tetrafluoro-1-methylcyclohexyl)methyl)terephthalamide (14)

Following analogous proceedure as in synthesis of **13**, tetrafluoro amine (**5b**) was used to furnish **14** (19 mg, 0.036 mmol, 48%) as a white crystalline solid; M.p. = 167 °C; ^{1}H NMR (400 MHz, CD_3CN) 7.92 (4H, s, H (Ar)), 7.13 (2H, t, *J* = 6.8, NH), 5.14-4.64 (4H, m, H- 2, 3, 5, 6), 3.81 (4H, d, *J* = 6.7, CH_2-NH), 2.54-2.35 (4H, m, H- 4), 0.93 (3H, t, *J* = 1.4, CH_3); ^{19}F NMR (376 MHz, CD_3CN) -195.5 (AA'XX'), -207.8 (s br); ^{13}C NMR (101 MHz, CD_3CN) 167.0 (COAr), 137.2 (C- (Ar)) , 127.4 (CH (Ar)) , 90.4 (d, *J* = 89.2, C- 2, 6), 85.9 (d, *J* = 196.2, C-3, 5), 42.4 (C-1), 41.2 (CH_2-NH), 27.5 (C-4), 15.6 (CH_3); FTMS (ESI$^+$) *m/z* calcd for ([M] + Na^+) 551.1920; found 551.1915; IR: v_{max}/cm^{-1} 3320 (N-H), 2924 (C-H), 1730 (C=O).

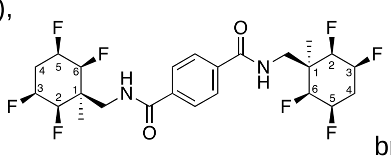

Bis-(2,3,5,6-tetrafluoro-(1-methylcyclohexyl)methyl) terephthalate (15)

Analogous esterification reaction has been done with tetephnaloyol chloride and 2,3,4,5-tetrafluoro alcohol, which has been previously reported [11], resulting in compound **15** (0.032 g, 0.06 mmol, 88%) as white solid; M.p. = 257 °C; ^{1}H NMR (500 MHz, $(CD_3)_2CO$): δ 8.20 (4H, s, Ar), 5.19 (4H, ddt, *J* = 47.6, 10.6, 3.1 Hz, H- 3, 5), 4.93 (4H, ddd, *J* = 44.3, 22.2, 3.2 Hz, H- 2, 6), 4.35 (4H, s, CH_2), 1.41 (6H, s, CH_3); ^{13}C NMR (126 MHz, $(CD_3)_2CO$): δ 164.7 (s, C(O)O), 133.9 (C-1'), 129.7 (C-2'), 88.8 (d, *J* = 183.9 Hz, C-3), 86.9 (d, *J* = 183.8 Hz, C-2), 65.0 (CH_2), 43.2 (s, C-4), 39.3 (s, C-1) 29.4 (s, CH_3); ^{19}F NMR (376 MHz, $(CD_3)_2CO$): δ -195.7 (s br, F-3), -209.4 (s br, F-2); FTMS (ESI$^-$) *m/z* calcd for ([M]+Cl^-) 565.1392; found 565.1396; IR: v_{max}/cm^{-1} 1728 (C=O), 1267, 1244 (C-O)

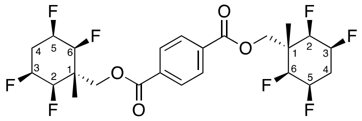

3. NMR Spectra

8a: methyl-dioxatricyclooctane-carbonitrile

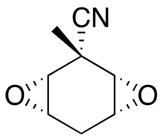

^{1}H NMR (400 MHz, $CDCl_3$)

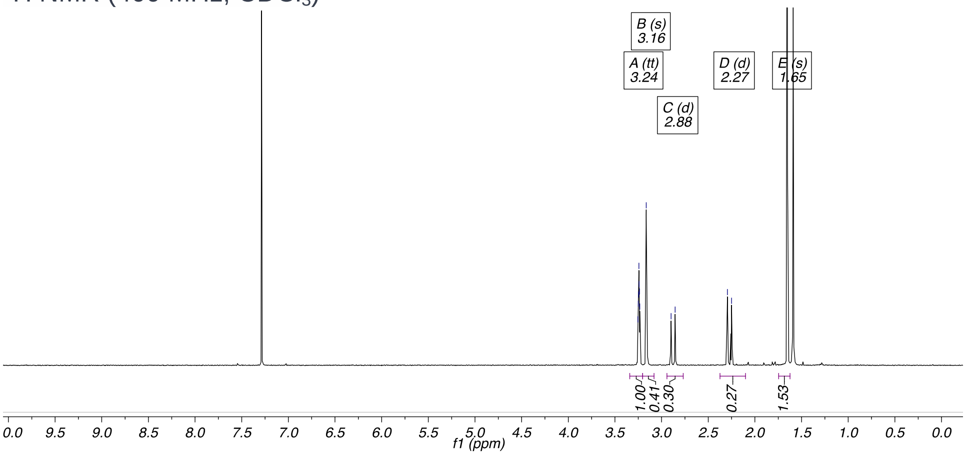

^{13}C NMR (101 MHz, $CDCl_3$)

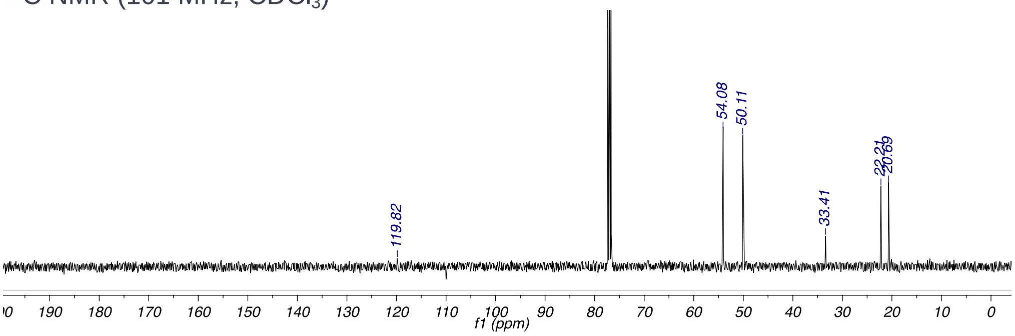

8b and 8c: methyl-dioxatricyclooctane-carbonitrile

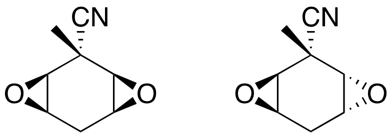

^{1}H NMR (400 MHz, $CDCl_3$)

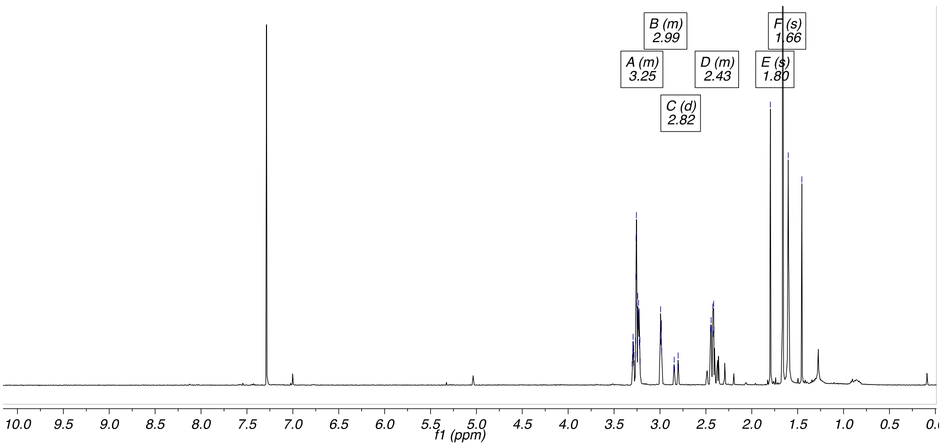

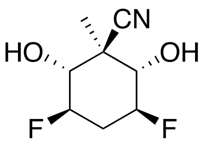

9a: difluoro-dihydroxy-methylcyclohexane-carbonitrile

^{1}H NMR (400 MHz, $CDCl_3$)

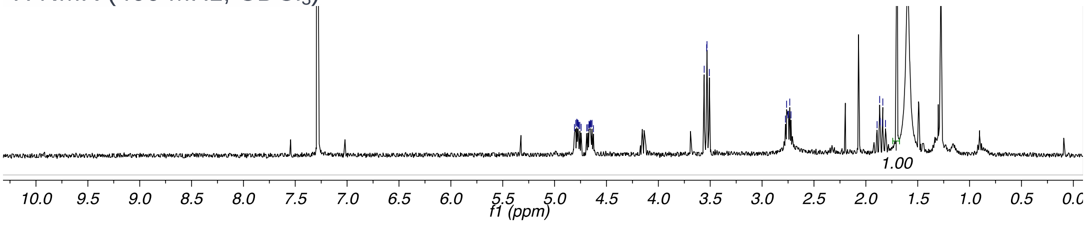

^{19}F NMR (376 MHz, $CDCl_3$)

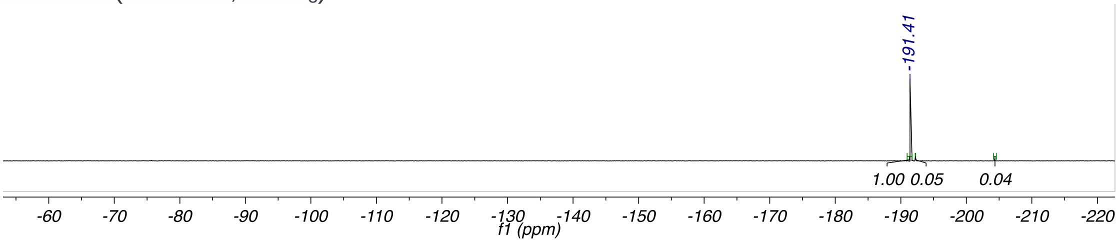

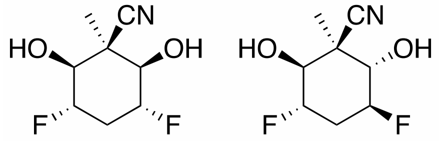

9b and 9c: difluoro-dihydroxy-methylcyclohexane-carbonitrile

^{1}H NMR (400 MHz, $CDCl_3$)

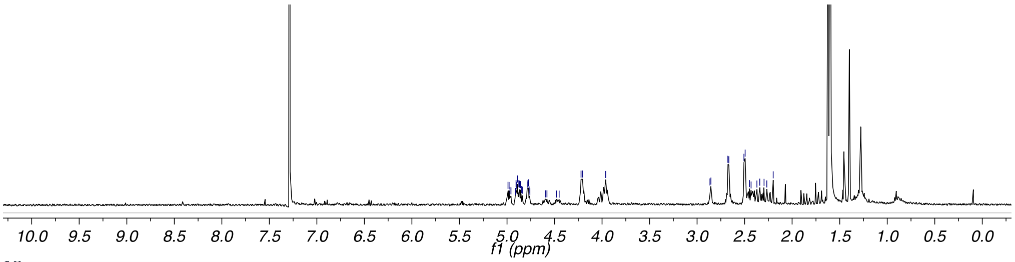

^{19}F NMR (376 MHz, $CDCl_3$)

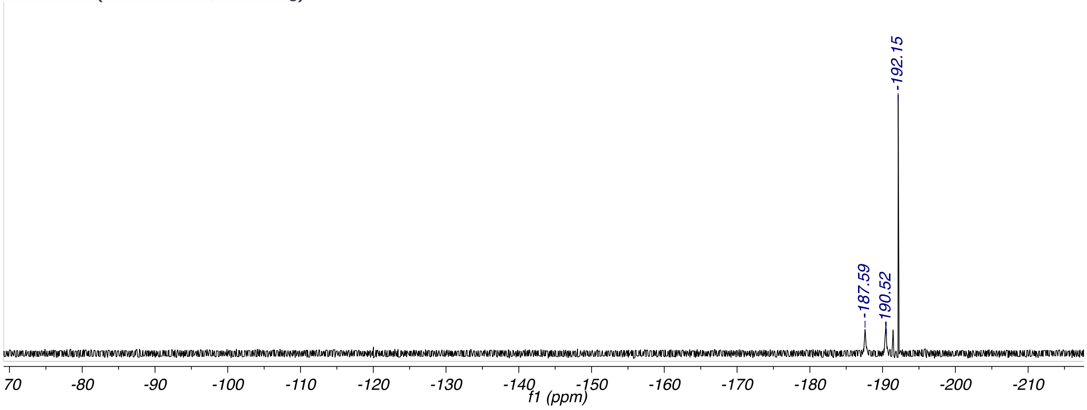

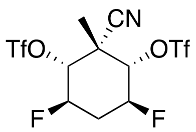

10a: cyano-difluoro-methylcyclohexane-diyl bis(trifluoromethanesulfonate

^{1}H NMR (400 MHz, $CDCl_3$)

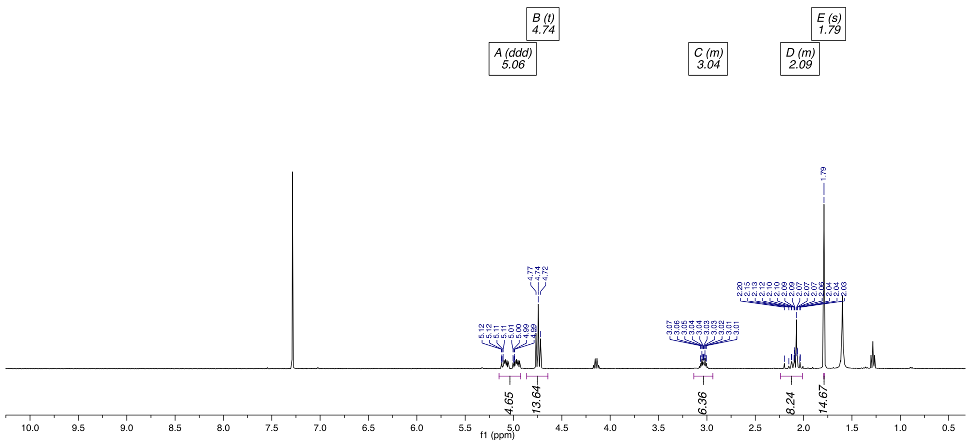

^{13}C NMR (101 MHz, $CDCl_3$)

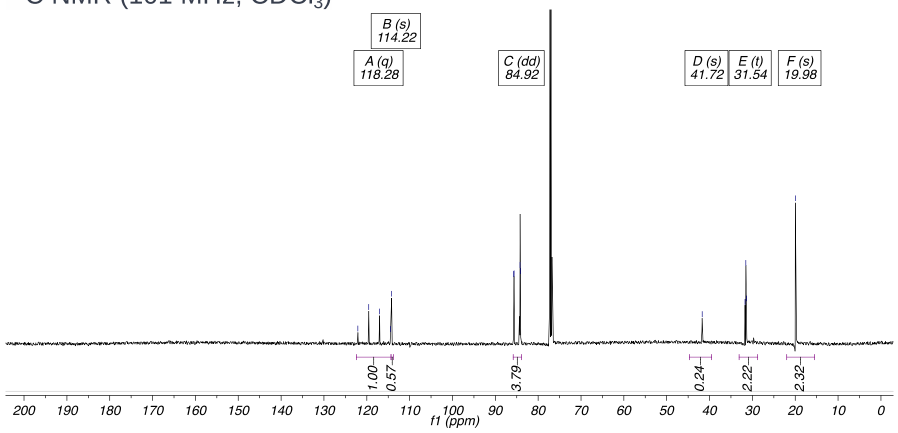

^{19}F NMR (376 MHz, $CDCl_3$)

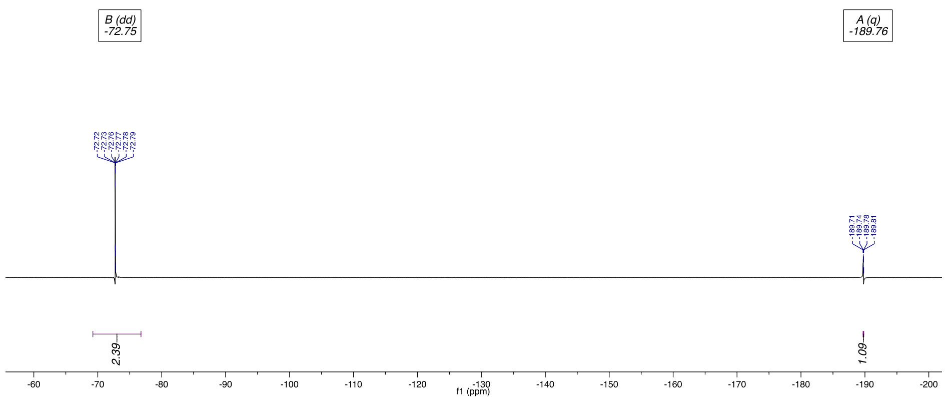

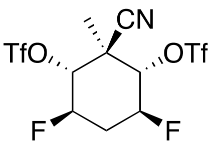

10b: cyano-difluoro-methylcyclohexane-diyl bis trifluoromethanesulfonate

^{1}H NMR (400 MHz, $CDCl_3$)

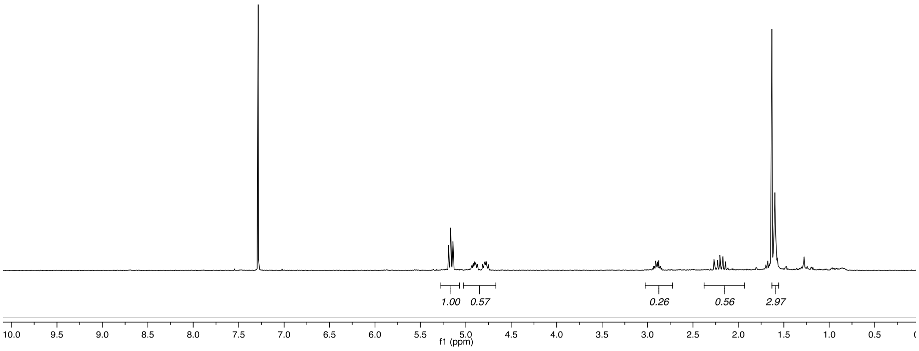

^{13}C NMR (101 MHz, $CDCl_3$)

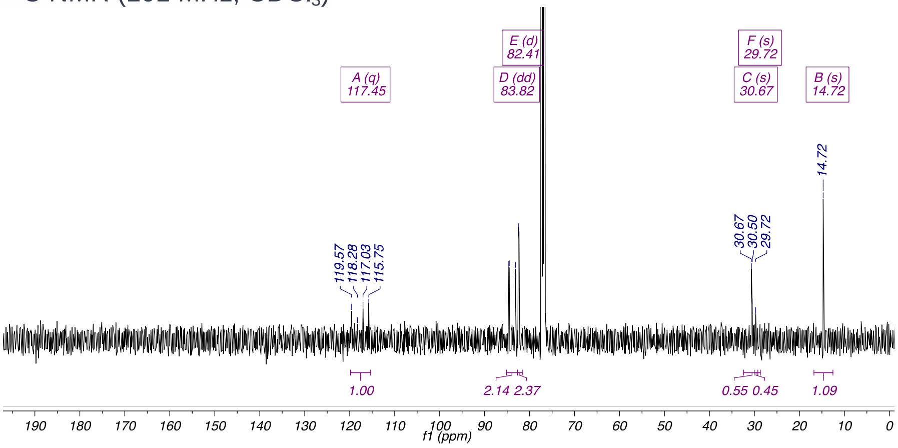

^{19}F NMR (376 MHz, $CDCl_3$)

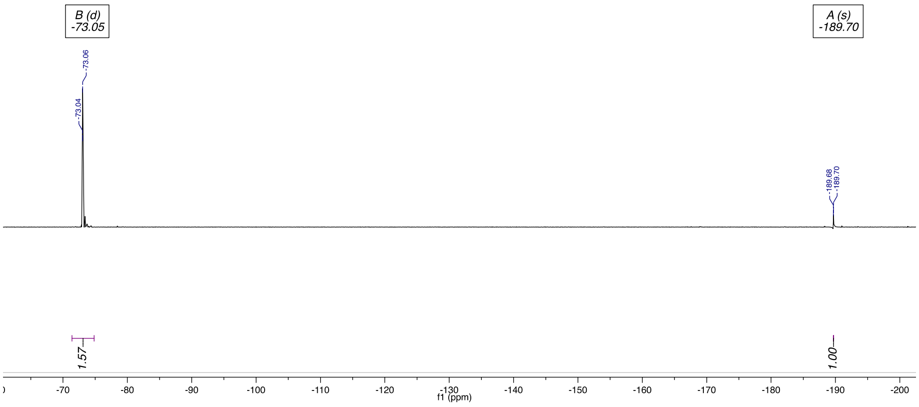

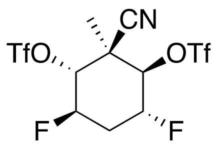

10c: cyano-difluoro-methylcyclohexane-diyl bis trifluoromethanesulfonate

^{1}H NMR (400 MHz, $CDCl_3$)

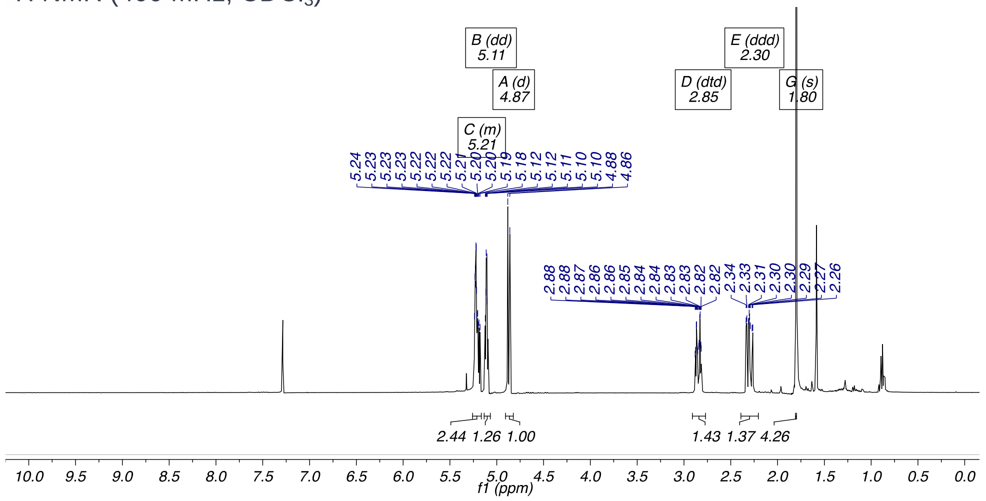

^{13}C NMR (101 MHz, $CDCl_3$)

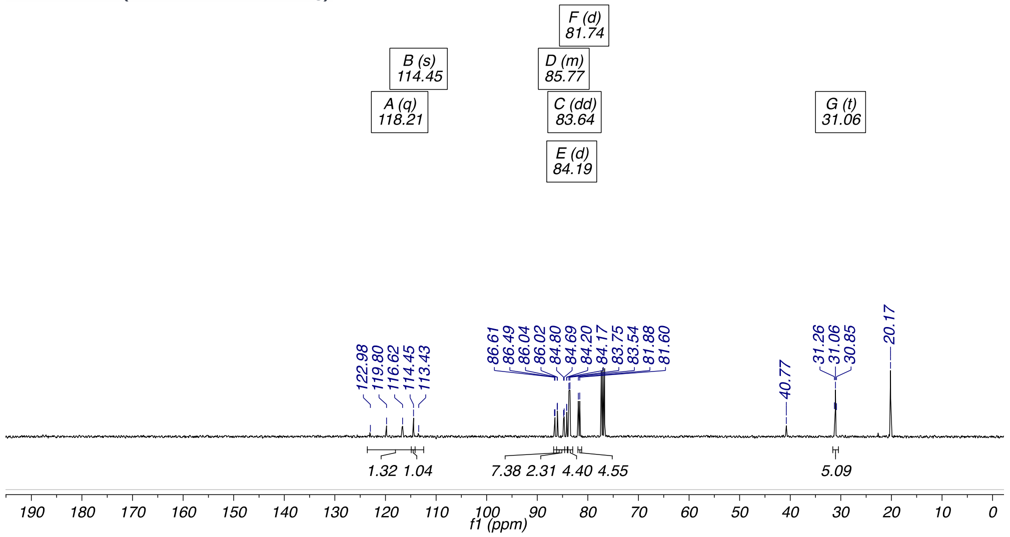

^{19}F NMR (376 MHz, $CDCl_3$)

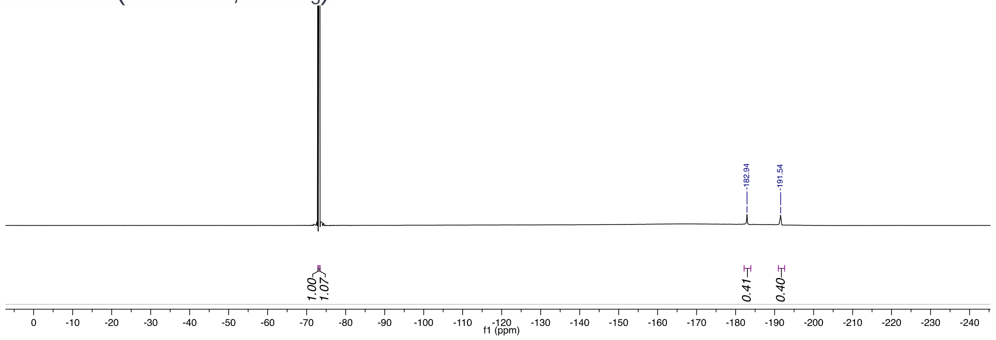

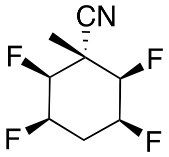

11a: 2,3,5,6-tetrafluoro-methylcyclohexane-carbonitrile

^{1}H NMR (400 MHz, $CDCl_3$)

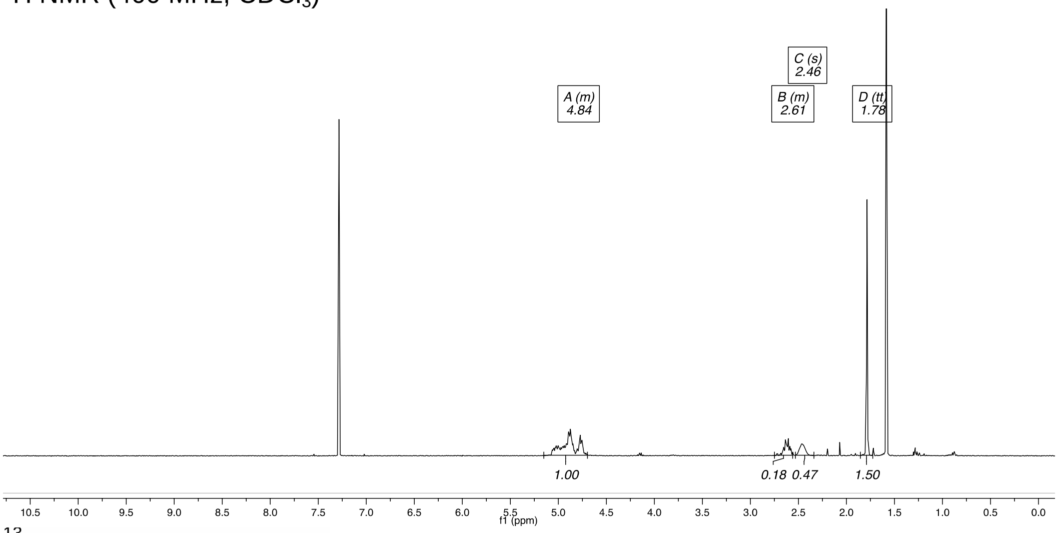

^{13}C NMR (126 MHz, $CDCl_3$)

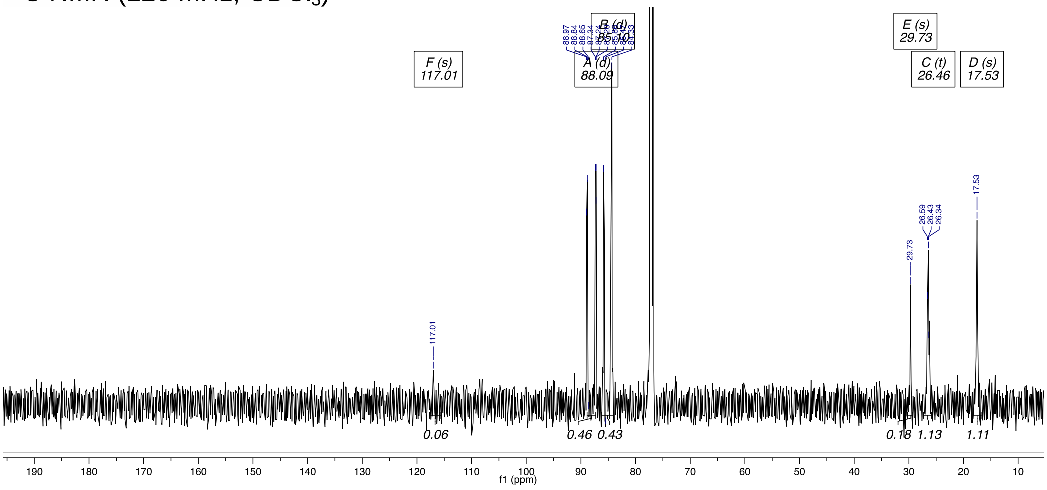

^{19}F NMR (376 MHz, $CDCl_3$)

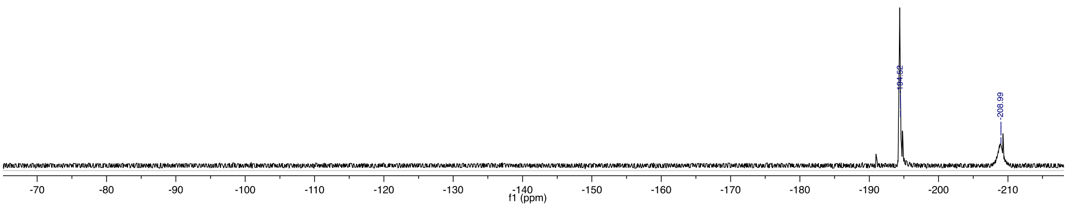

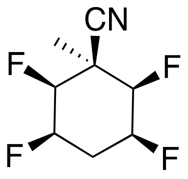

11b: 2,3,5,6-tetrafluoro-1-methylcyclohexane-carbonitrile

^{1}H NMR (400 MHz, $CDCl_3$)

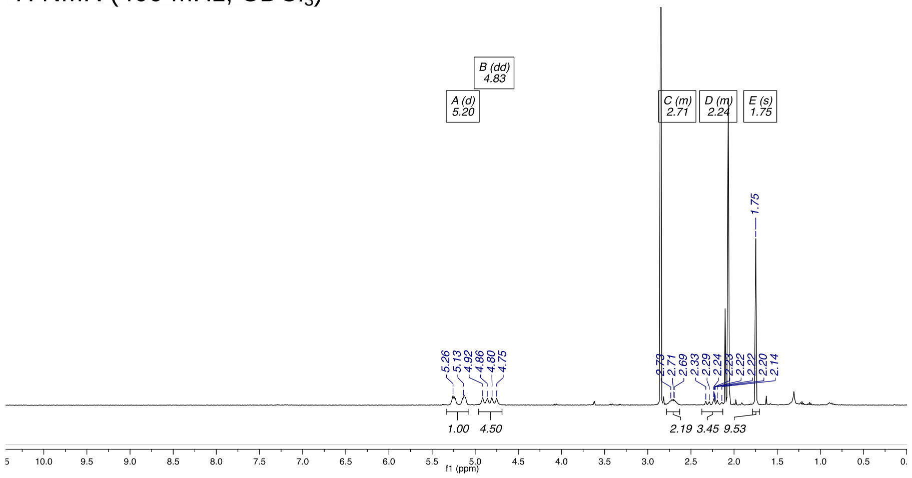

^{13}C NMR (126 MHz, $CDCl_3$)

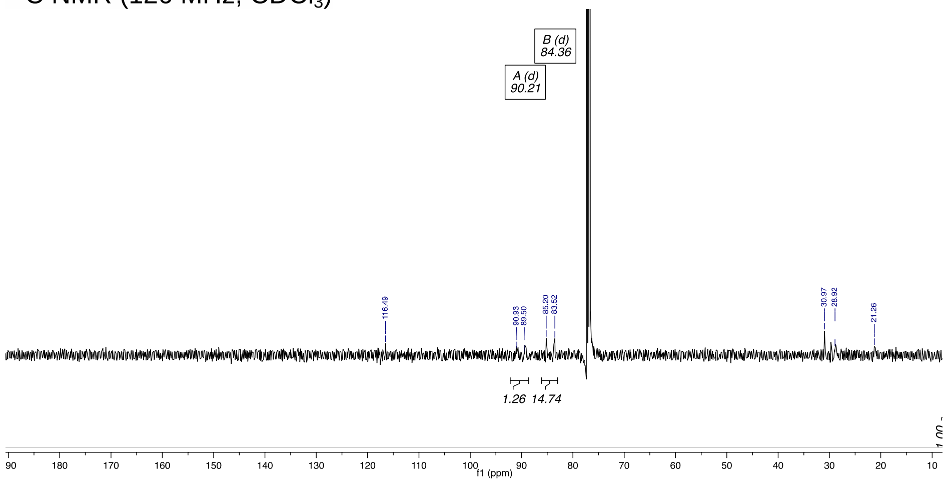

^{19}F NMR (376 MHz, $CDCl_3$)

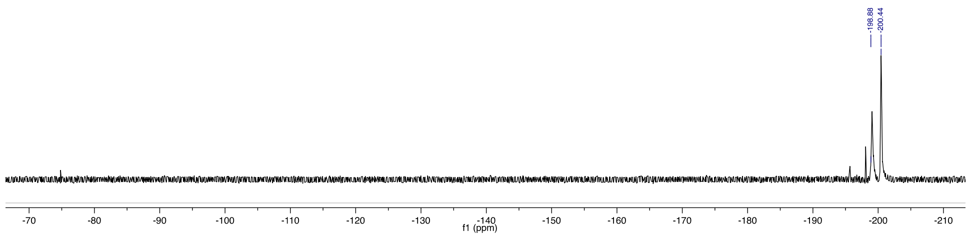

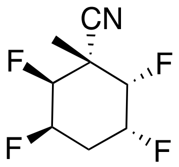

11c: 2,3,5,6-tetrafluoro-1-methylcyclohexane-1-carbonitrile

^{1}H NMR (400 MHz, $CDCl_3$)

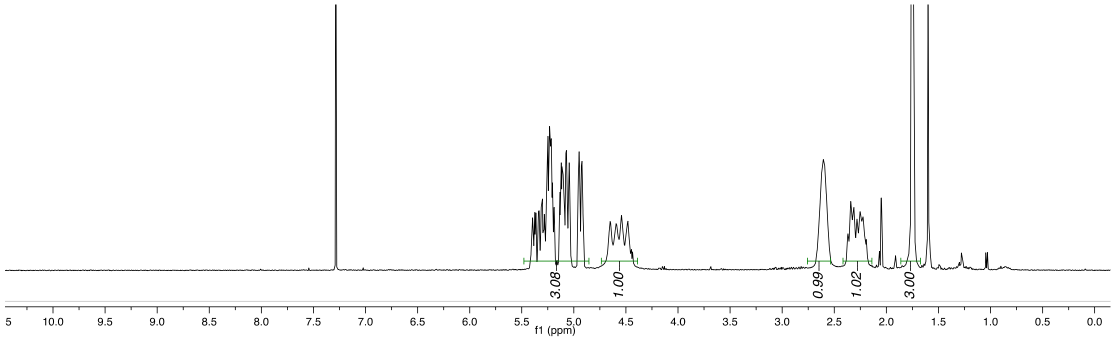

^{13}C NMR (126 MHz, $CDCl_3$)

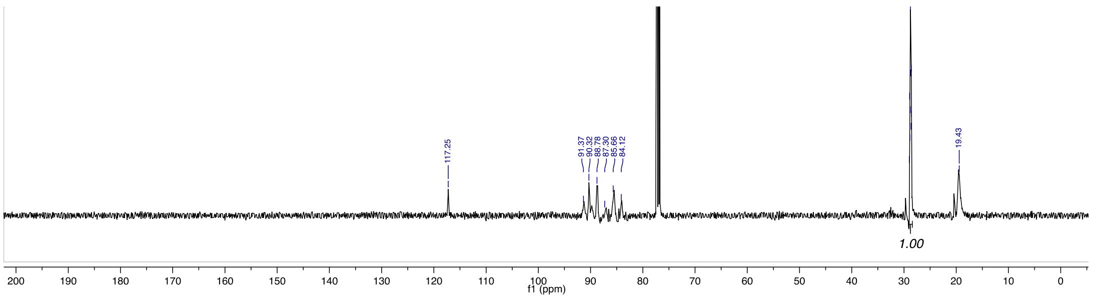

^{19}F NMR (376 MHz, $CDCl_3$)

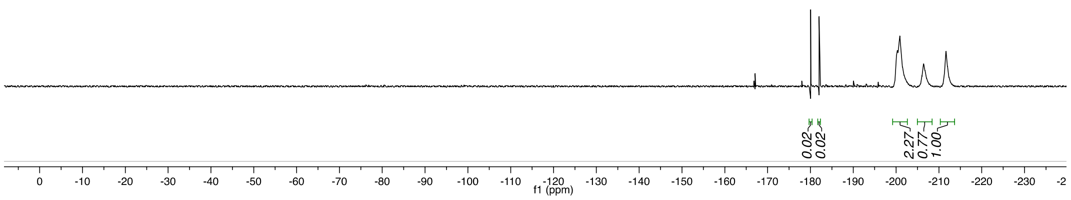

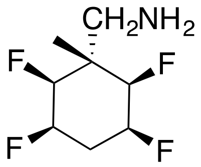

5a: 2,3,5,6-tetrafluoro-methylcyclohexyl)methanamine

^{1}H NMR (400 MHz, MeOD)

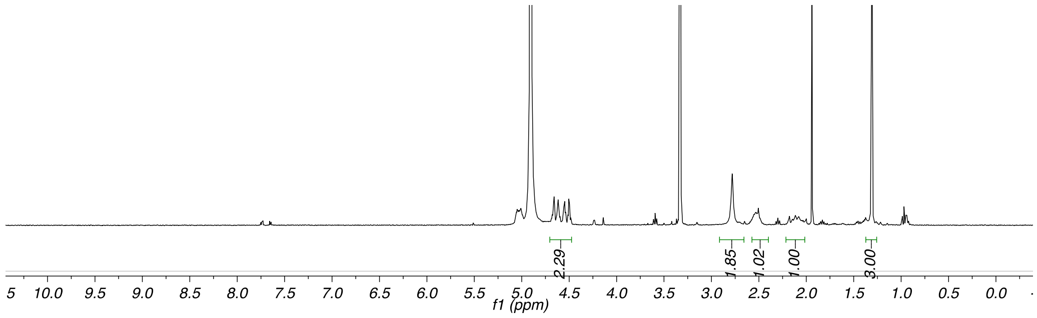

^{13}C NMR (126 MHz, MeOD)

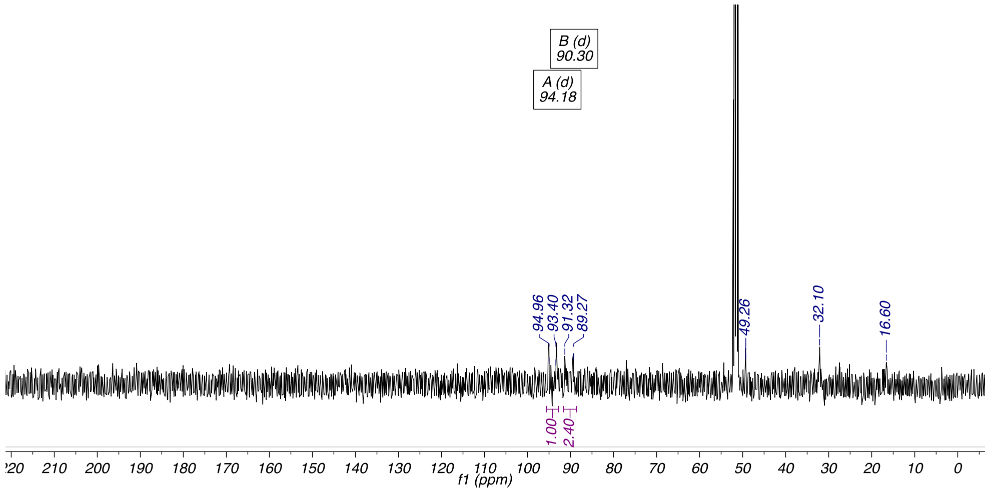

^{19}F NMR (377 MHz, MeOD)

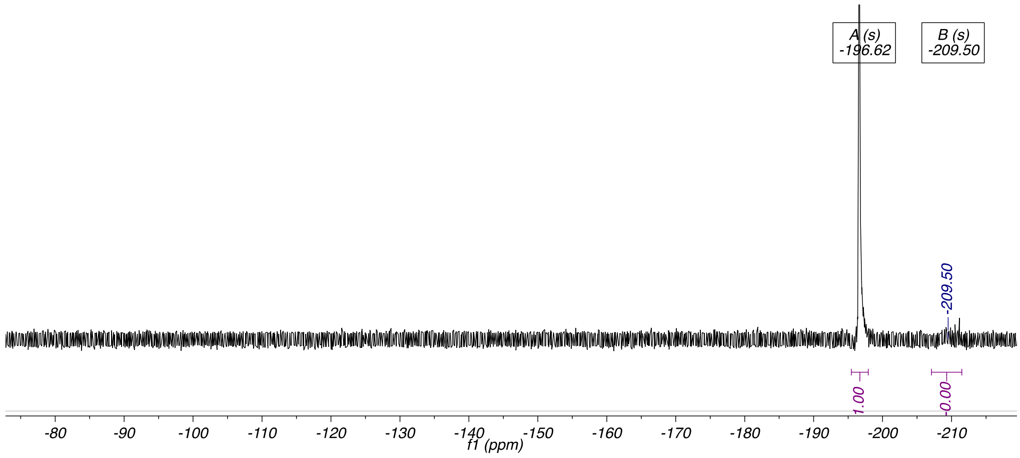

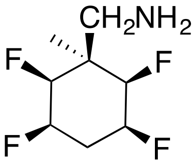

5b: 2,3,5,6-tetrafluoro-methylcyclohexyl methanamine

^{1}H NMR (400 MHz, MeOD)

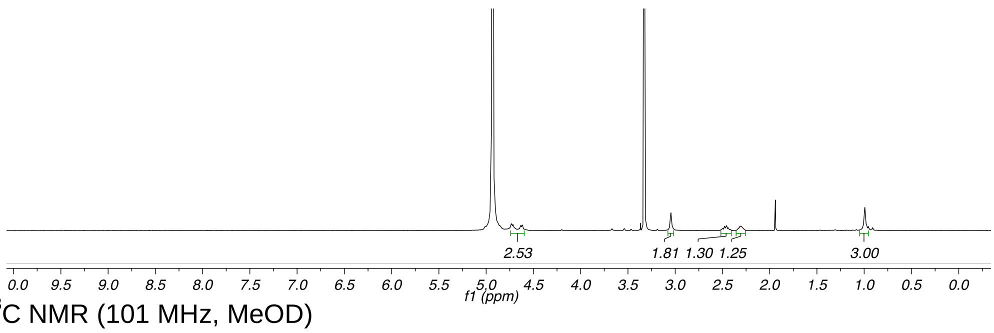

^{13}C NMR (101 MHz, MeOD)

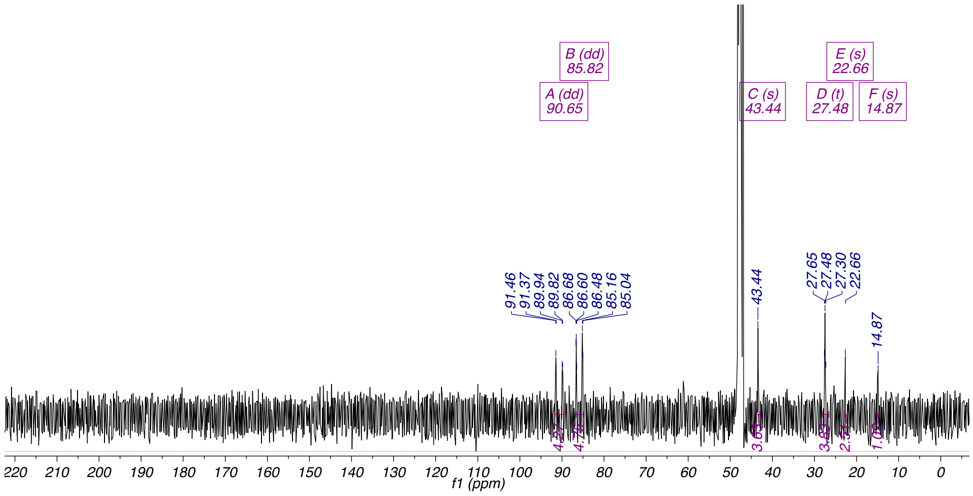

^{19}F NMR (376 MHz, MeOD)

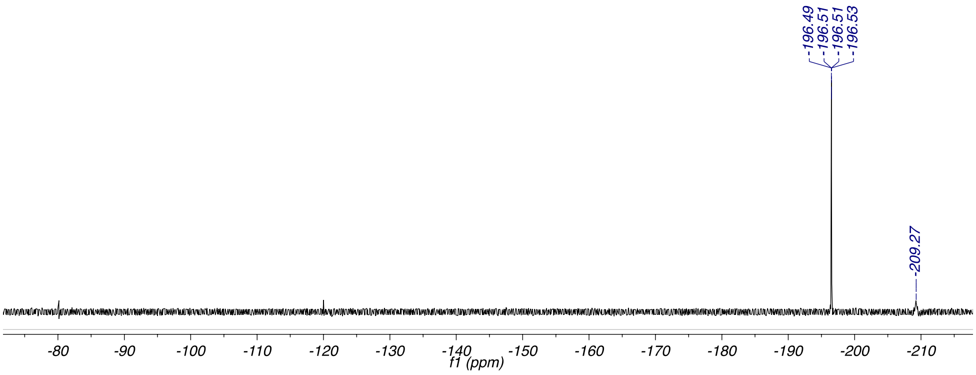

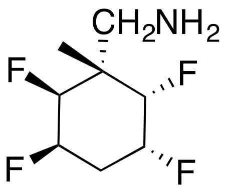

5c: 2,3,5,6-tetrafluoro-methylcyclohexyl methanamine

^{1}H NMR (400 MHz, MeOD)

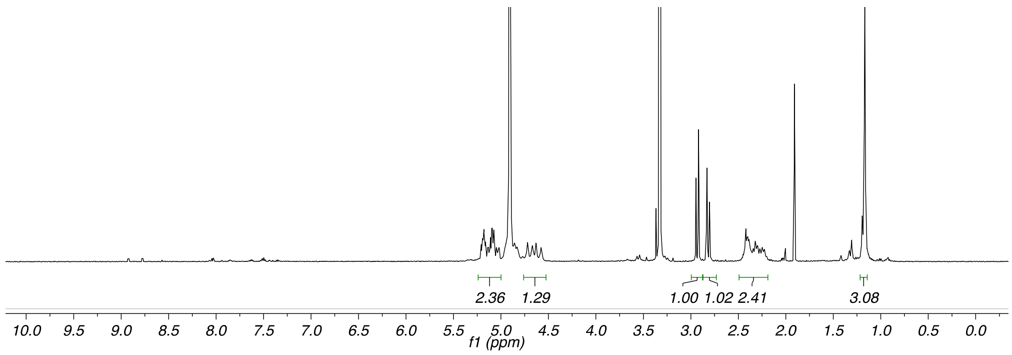

^{13}C NMR (126 MHz, MeOD)

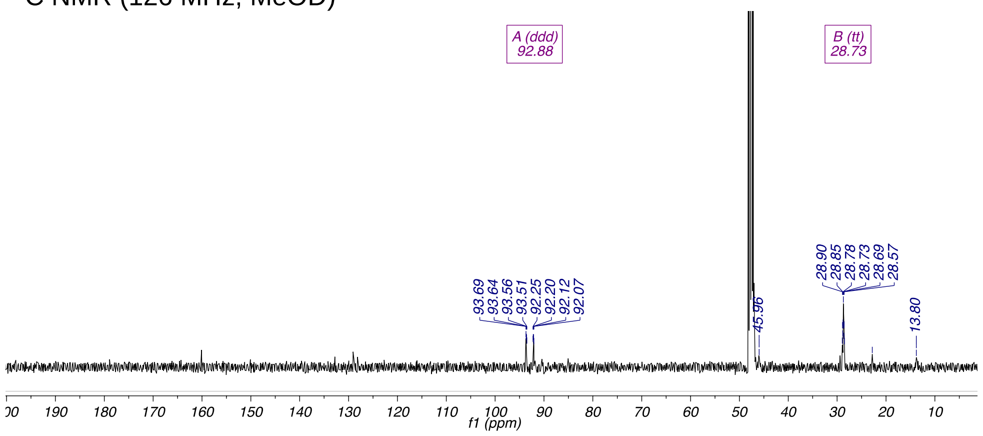

^{19}F NMR (377 MHz, MeOD)

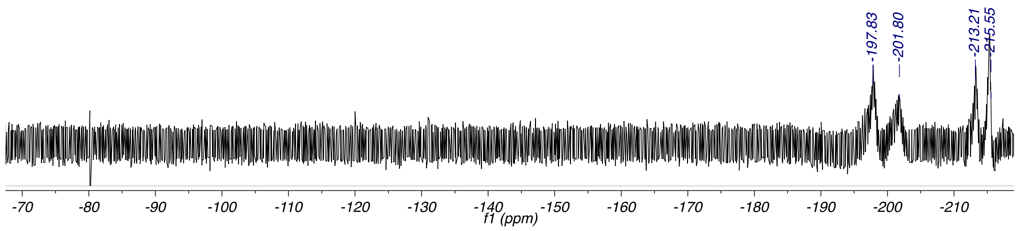

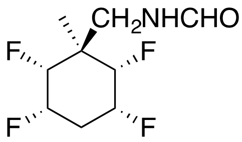

12: 2,3,5,6-tetrafluoro-1-methylcyclohexyl)methyl)formamide

^{1}H NMR (400 MHz, $(CD_3)_2CO$)

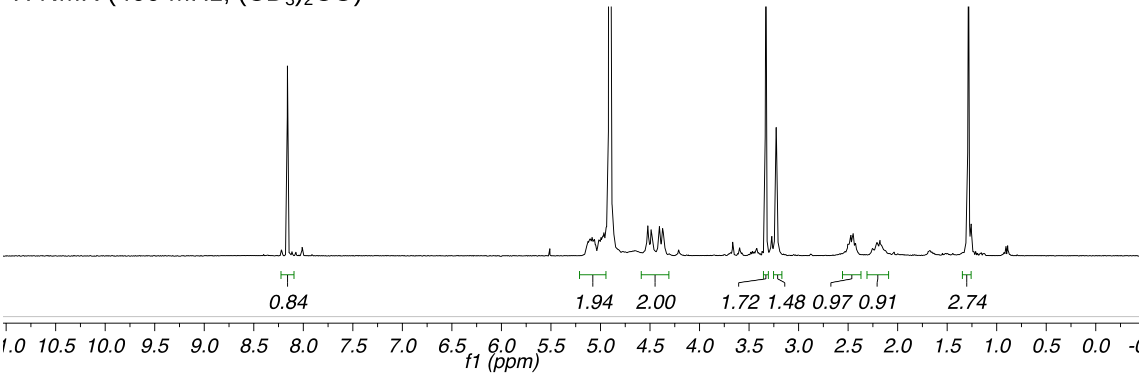

^{13}C (126 MHz, $(CD_3)_2CO$)

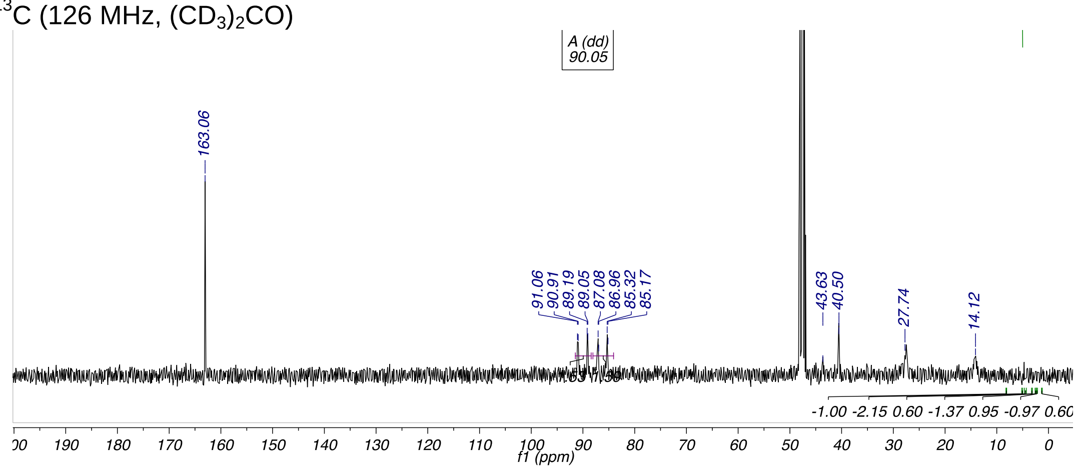

^{19}F (282 MHz, $(CD_3)_2CO$)

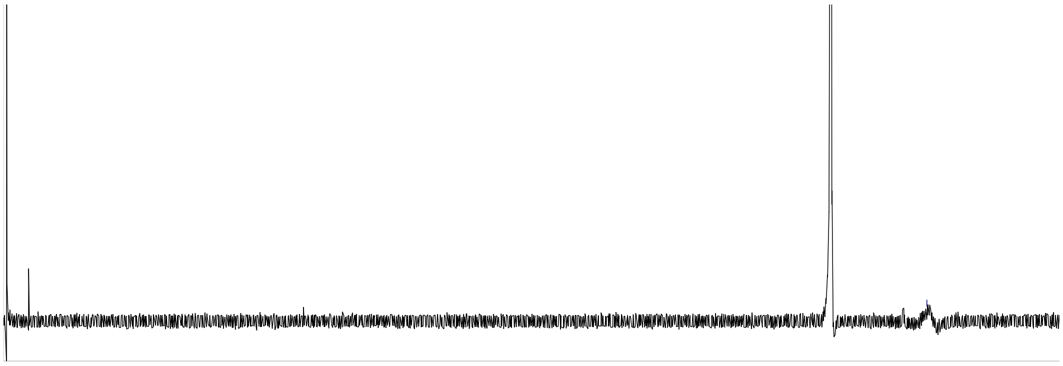

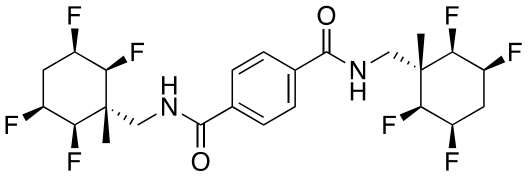

13: 2,3,5,6-tetrafluoro-methylcyclohexyl methyl terephthalamide

^{1}H NMR (400 MHz, $(CD_3)_2CO$)

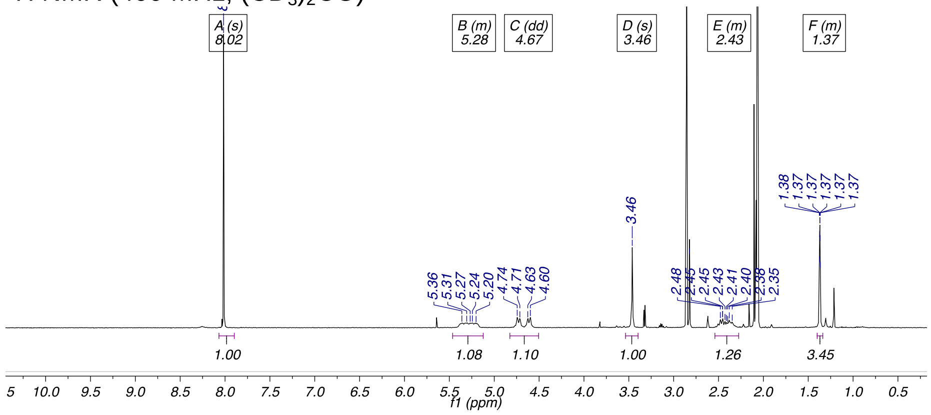

^{13}C (126 MHz, $(CD_3)_2CO$)

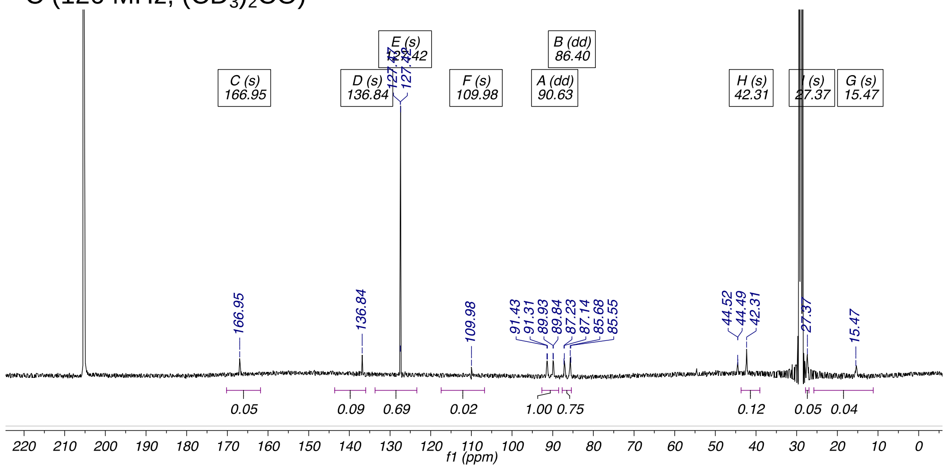

^{19}F NMR (376 MHz, $(CD_3)_2CO$)

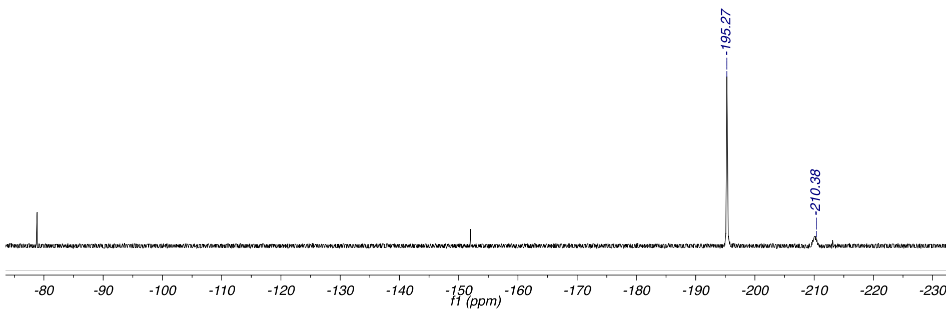

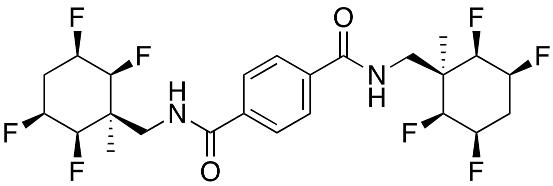

14: 2,3,5,6-tetrafluoro-methylcyclohexyl methyl terephthalamide

^{1}H NMR (400 MHz, CD_3CN)

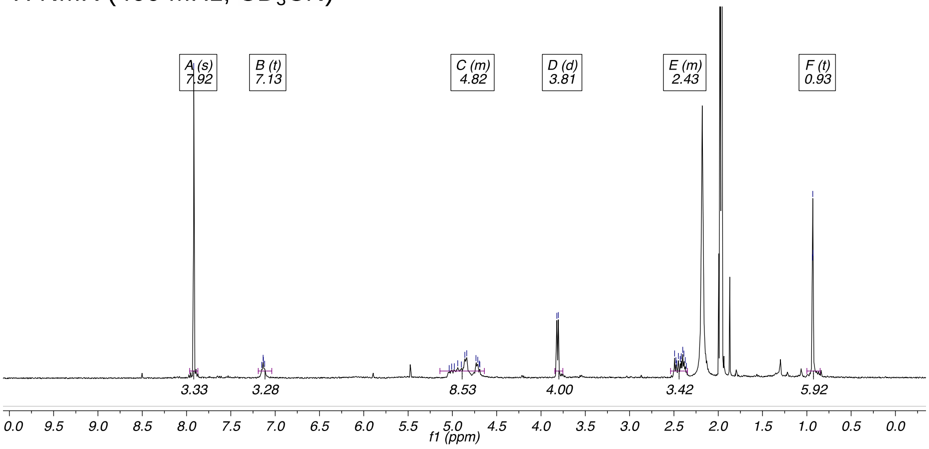

^{13}C NMR (101 MHz, CD_3CN)

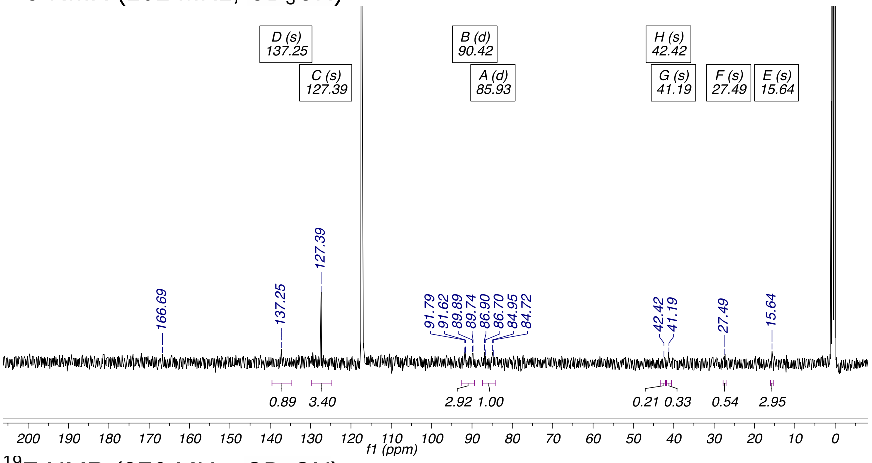

^{19}F NMR (376 MHz, CD_3CN)

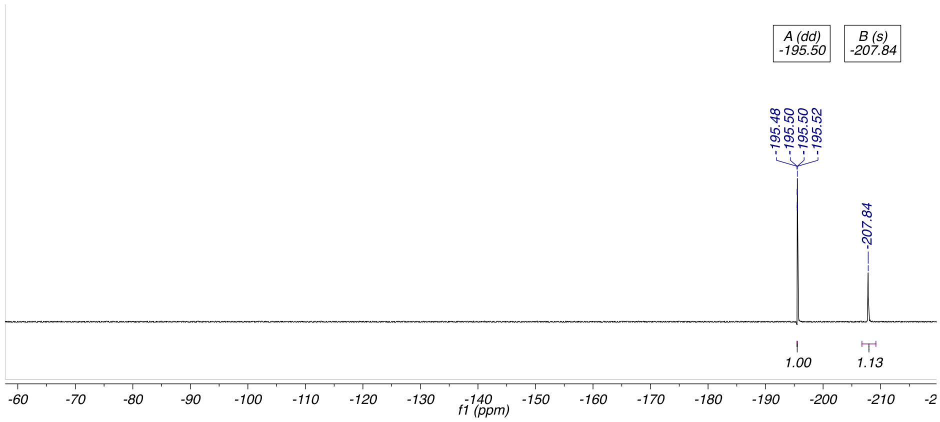

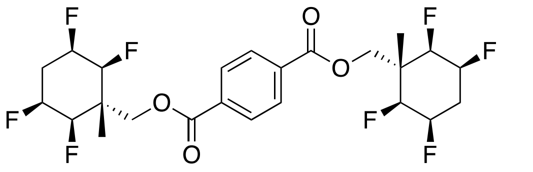

15*:* 2,3,5,6-tetrafluoro-methylcyclohexyl methyl terephthalamide

^{1}H NMR (400 MHz, $(CD_3)_2CO$)

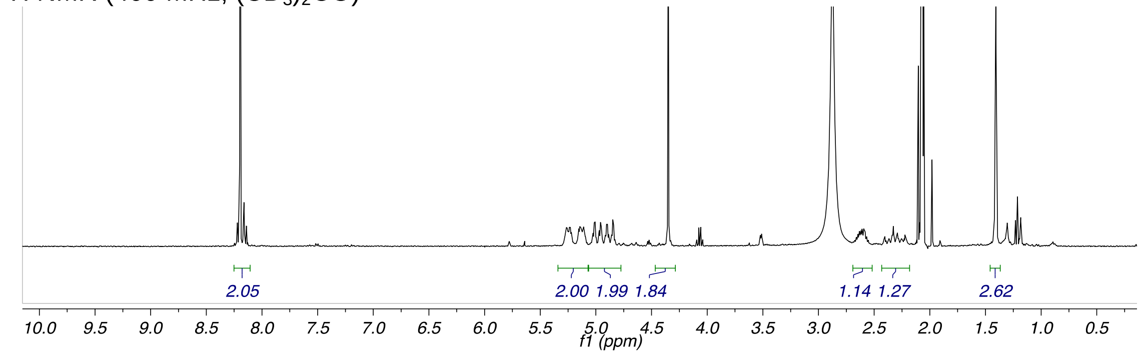

^{13}C (126 MHz, $(CD_3)_2CO$)

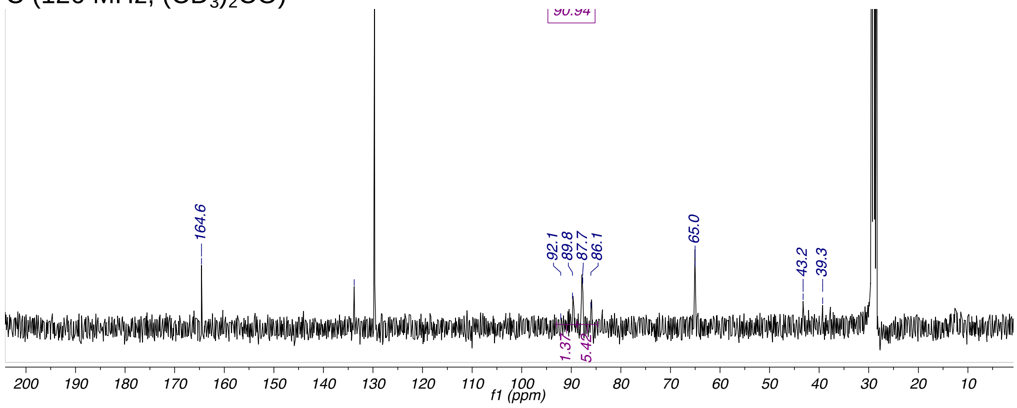

^{19}F (282 MHz, $(CD_3)_2CO$)

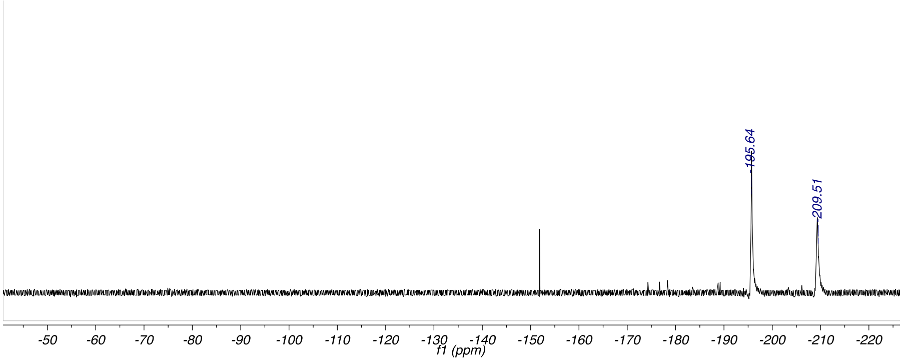

Supplement: File 1 — Experimental part. [file Beilstein_J_Org_Chem-13-728-s001.pdf]
